# Fused ultrasound and electromyography-driven neuromuscular model to improve plantarflexion moment prediction across walking speeds

**DOI:** 10.1186/s12984-022-01061-z

**Published:** 2022-08-09

**Authors:** Qiang Zhang, Natalie Fragnito, Jason R. Franz, Nitin Sharma

**Affiliations:** 1 Joint Department of Biomedical Engineering at the University of North Carolina-Chapel Hill and North Carolina State University, 1840 Entrepreneur Dr., 27695 Raleigh, NC USA; 2 Joint Department of Biomedical Engineering at the University of North Carolina-Chapel Hill and North Carolina State University, 333 S Columbia St., 27514 Chapel Hill, NC USA

**Keywords:** Human intent prediction, Neuromuscular model, B-mode ultrasound imaging, Surface electromyography, Rehabilitative/assistive robotics, Sensor fusion

## Abstract

**Background:**

Improving the prediction ability of a human-machine interface (HMI) is critical to accomplish a bio-inspired or model-based control strategy for rehabilitation interventions, which are of increased interest to assist limb function post neurological injuries. A fundamental role of the HMI is to accurately predict human intent by mapping signals from a mechanical sensor or surface electromyography (sEMG) sensor. These sensors are limited to measuring the resulting limb force or movement or the neural signal evoking the force. As the intermediate mapping in the HMI also depends on muscle contractility, a motivation exists to include architectural features of the muscle as surrogates of dynamic muscle movement, thus further improving the HMI’s prediction accuracy.

**Objective:**

The purpose of this study is to investigate a non-invasive sEMG and ultrasound (US) imaging-driven Hill-type neuromuscular model (HNM) for net ankle joint plantarflexion moment prediction. We hypothesize that the fusion of signals from sEMG and US imaging results in a more accurate net plantarflexion moment prediction than sole sEMG or US imaging.

**Methods:**

Ten young non-disabled participants walked on a treadmill at speeds of 0.50, 0.75, 1.00, 1.25, and 1.50 m/s. The proposed HNM consists of two muscle-tendon units. The muscle activation for each unit was calculated as a weighted summation of the normalized sEMG signal and normalized muscle thickness signal from US imaging. The HNM calibration was performed under both single-speed mode and inter-speed mode, and then the calibrated HNM was validated across all walking speeds.

**Results:**

On average, the normalized moment prediction root mean square error was reduced by 14.58 % ($p=0.012$) and 36.79 % ($p<0.001$) with the proposed HNM when compared to sEMG-driven and US imaging-driven HNMs, respectively. Also, the calibrated models with data from the inter-speed mode were more robust than those from single-speed modes for the moment prediction.

**Conclusions:**

The proposed sEMG-US imaging-driven HNM can significantly improve the net plantarflexion moment prediction accuracy across multiple walking speeds. The findings imply that the proposed HNM can be potentially used in bio-inspired control strategies for rehabilitative devices due to its superior prediction.

**Supplementary Information:**

The online version contains supplementary material available at 10.1186/s12984-022-01061-z.

## Background

Locomotion mobility accounts for a dominant part of human activities of daily living, like moving around the home and community, going to work or school, doing errands, visiting friends, etc. The human lower extremity plays an essential role in achieving locomotion mobility. The human ankle plantarflexors generate a large burst of “push-off” mechanical power during the late stance phase of walking, enabling forward and upward acceleration of the body’s center of mass. Due to neurological disorders or injuries like spinal cord injury, stroke, and multiple sclerosis, the weakened function or dysfunction of plantarflexors is likely to cause a dramatic decrease in the “push-off” power. Consequently, these mobility disorders impair walking function and cause poor energy economy [1], as well as disrupt both physical and emotional well-being [2].

Modern neurorehabilitation devices, such as powered ankle exoskeletons [3–5], soft exosuits [6, 7], and functional electrical stimulation [8–12], may use assist-as-needed control to actively engage and maximize recovery of users with mobility impairments [13, 14]. In turn, the efficacy of the control strategy depends on the accurate determination of continuous human volitional movement intent (net joint moment). Mechanical sensors, like force or torque sensors, installed on a rigid and bulky frame, have been often used to measure the human intent for joints without direct interaction to the ground, but limit the system’s wearability. Also, inaccuracies may creep in easily due to the inevitable misalignment between the bionic joint center and human joint center, which may introduce undesired interaction force [15, 16]. Usually, it is challenging to directly measure the net ankle joint moment during walking overground with conventional force or torque sensors setup. The standard way to measure the net ankle joint moment uses a motion capture system, ground reaction force (GRF), and inverse dynamics (ID) calculations. However, there are two shortcomings of the standard approach. First, the setup is constrained to a lab environment, and not applicable for field testing. Second, the results from ID do not reveal how skeletal muscles perform during walking from a neuromuscular perspective. Therefore, a forward dynamics approach based on a neuromuscular model would be very instrumental when a motion capture system and GRF data are unavailable.

Surface electromyography (sEMG) measures electrical potentials during asynchronous muscle neurons firings, and its amplitude and frequency positively relate to muscle activation levels. Therefore, sEMG-derived signals can be used in a Hill-type neuromuscular model (HNM) or to train a machine learning approach (model-free) to predict volitional joint moment [17, 18] and angular position [19–21]. However, sEMG signals suffer from interference or cross-talking from the adjacent muscles, and an inability of measuring activations of deep-layer muscles [22, 23]. Alternatively, two-dimensional brightness mode (B-mode) ultrasound (US) imaging allows one to see the musculature of the targeted muscle in vivo. Due to its ability to directly visualize superficial and deep-layer muscles, US imaging may work as an alternative methodology to predict joint motion or motion intent. Potentially, US imaging overcomes the shortcomings of the sEMG measurements. Most frequently used architectural features from US images include pennation angle (PA) [24, 25], fascicle length (FL) [26, 27], muscle thickness (MT) [28, 29], and cross-sectional area [30]. These features have been correlated with the joint kinetics and kinematics during isometric or isokinetic joint motion by using HNM-based or model-free approaches [25, 31–33].

Motivation also exists to use a dual-modal approach that combines the measurement from sEMG and US imaging. Potentially sEMG signals and US imaging provide complementary information, and the combination between them may (1) mitigate any cross-talking effect from neighboring sEMG signals and (2) lower US imaging-derived features’ drift due to the accumulated errors from cyclic joint movement. Our recent studies have shown the advantages of using the dual-modal approach over uni-modal bio-signals (sEMG or US imaging) for ankle joint moment/motion prediction under isometric/dynamic dorsiflexion studies [21, 25, 27] and isometric plantarflexion study [34, 35]. Similarly, Dick et al. used both sEMG and US imaging to predict plantarflexion force during dynamic cycling tasks [32]. However, the use of the dual-modal bio-signals during more complex functional tasks, like walking across different speeds remains unexplored.

In this study, we investigated a dual-modal approach that takes both processed sEMG and MT from US imaging as inputs to a modified HNM, named sEMG-US imaging-driven HNM, to predict net ankle joint plantarflexion moment during the walking stance phase across multiple speeds. We hypothesize that (1) the proposed HNM will achieve a better net plantarflexion moment prediction accuracy than sEMG-driven and US imaging-driven HNMs, (2) the net plantarflexion moment prediction performance is more robust if data collected from multiple speeds are included in HNMs’ calibration procedure.

In previous US imaging studies, visualized architectural features, such as PA and FL, require high-resolution US imaging, which can be significantly affected by a US transducer placement site on the muscle. To mitigate the requirement of high-resolution US imaging, US imaging-derived signals such as echogenicity [27, 36, 37], tissue displacement [38], and MT [29], are more preferable to correlate with muscle or joint mechanical functions. Because ankle plantarflexors: lateral and medial gastrocnemius and soleus muscles (LGS, MGS, and SOL) are not accessible in the same US image plane, this study chose to focus on LGS and SOL, which are in the same plane, and tracked their MT change during the walking experiments across multiple speeds. Therefore, the contributions of the paper are: (1) the use of US imaging-derived MT changes and sEMG-derived changes of LGS and SOL muscles as surrogate variables of muscle activations during walking, (2) development of a modified HNM that uses both sEMG and US imaging as inputs for single-speed modes and an inter-speed mode, and (3) evaluation of the sEMG-US imaging-driven HNM’s robustness across multiple walking speeds.

## Method

### Hill-type neuromuscular model of ankle joint

Below, we propose a modified sEMG-US imaging-driven HNM to directly build a relationship between the joint net PF moment and sEMG-US imaging-derived surrogate signals of both LGS and SOL muscles. There are three sub-models involved in the HNM: (a) sEMG-US imaging-derived weighted muscle activation model, (b) muscle-tendon unit geometry model, and (c) muscle contraction dynamic model.

#### Weighted muscle activation model

The neural activation at $t_{k}$ time instant $N_{i}(t_{k}),\,i=1,2$, $k=1,2,3,...$, for SOL and LGS muscles, respectively, considers the electromechanical delay (EMD), $\tau$, between the onset of an sEMG signal and a muscle contraction and utilizes a second-order recursive filter and is defined as [39]

1 $$\begin{aligned} N_{i}(t_{k})=\alpha _{i}u_{i}(t_{k-\tau })-\beta _{1i}N_{i}(t_{k-1})-\beta _{2i}N_{i}(t_{k-2}) \end{aligned}$$

where EMD, $\tau$, is usually between 30 ms and 120 ms. $u_{i}(t_{k})$ represents the sEMG’s linear envelope normalized to the peak value of the specific task (with a constant peak value across all walking speeds on each subject in this study). The sEMG’s linear envelope was derived after raw sEMG signals’ band-pass filtering, full-wave rectification, and low-pass filtering. $\alpha _{i},$ $\beta _{1i}$, and $\beta _{2i}$ are coefficients that define the recursive filter’s dynamics of each muscle [39], and the following set of constrains is employed to reach a positive stable solution, i.e.

2 $$\begin{aligned} \beta _{1i}=\gamma _{1i}+\gamma _{2i},\,\beta _{2i}=\gamma _{1i}\cdot \gamma _{2i},\,\alpha _{i}-\beta _{1i}-\beta _{2i}=1 \end{aligned}$$

where $$\left| \gamma _{1i}\right| <1$$ and $$\left| \gamma _{2i}\right| <1$$.

A nonlinear relationship between neural activation $N_{i}(t_{k})$ and corresponding muscle activation $a_{1i}(t_{k})$ is given as [39]

3 $$\begin{aligned} a_{1i}=\frac{e^{A_{i}N_{i}}-1}{e^{A_{i}}-1} \end{aligned}$$

where $A_{i}$ represents the nonlinear shape factor for each muscle, which is allowed to vary between − 3 and 0, with $A_{i}=-3$ being a nonlinear and $A_{i}=0$ being a linear relationship.

According to the reported results in [40–43], the MT change was found to correlate with the muscle contraction level or muscle activation through a linear function or piece-wise linear function. Therefore, in this work, the second part of the LGS or SOL muscle activation is calculated from the US imaging-derived MT change. The MT values of LGS and SOL muscles are denoted as $MT_{i}(t_{k})$. According to the preliminary results in [44], there is a positive relationship between MT change and the targeted muscle contraction level during the walking stance phase. After taking normalization of the MT with respect to the lower bound (at rest state) and upper bound (peak value of the specific task) on each participant, the US imaging-derived muscle activation, $a_{2i}(t_{k})$, is proposed as

4 $$\begin{aligned} a_{2i}=\frac{MT_{i}-MT_{i\min }}{MT_{i\max }-MT_{i\min }} \end{aligned}$$

where $MT_{i\max }$ and $MT_{i\min }$ represent the constant subjective MT values when the LGS and SOL muscles are at the walking task-specific maximum voluntary contraction and complete rest condition, respectively. For each individual, $MT_{i\max }$ and $MT_{i\min }$ are set as consistent values across different walking speeds. This normalization guarantees that the US imaging-derived muscle activations vary between 0 and 1.

By introducing an allocation gain between the sEMG- and US imaging-derived muscle activations, the synthesized/weighted muscle activation levels for LGS or SOL muscle, $a_{i}(t_{k})$, can be represented as

5 $$\begin{aligned} a_{i}=\delta _{i}a_{1i}+(1-\delta _{i})a_{2i} \end{aligned}$$

where $\delta _{i}\in [0,\,1]$ represents the muscle activation allocation gain for LGS ($i=1$) and SOL ($i=2$) muscles.

#### Muscle-tendon unit geometry model

**Fig. 1:**
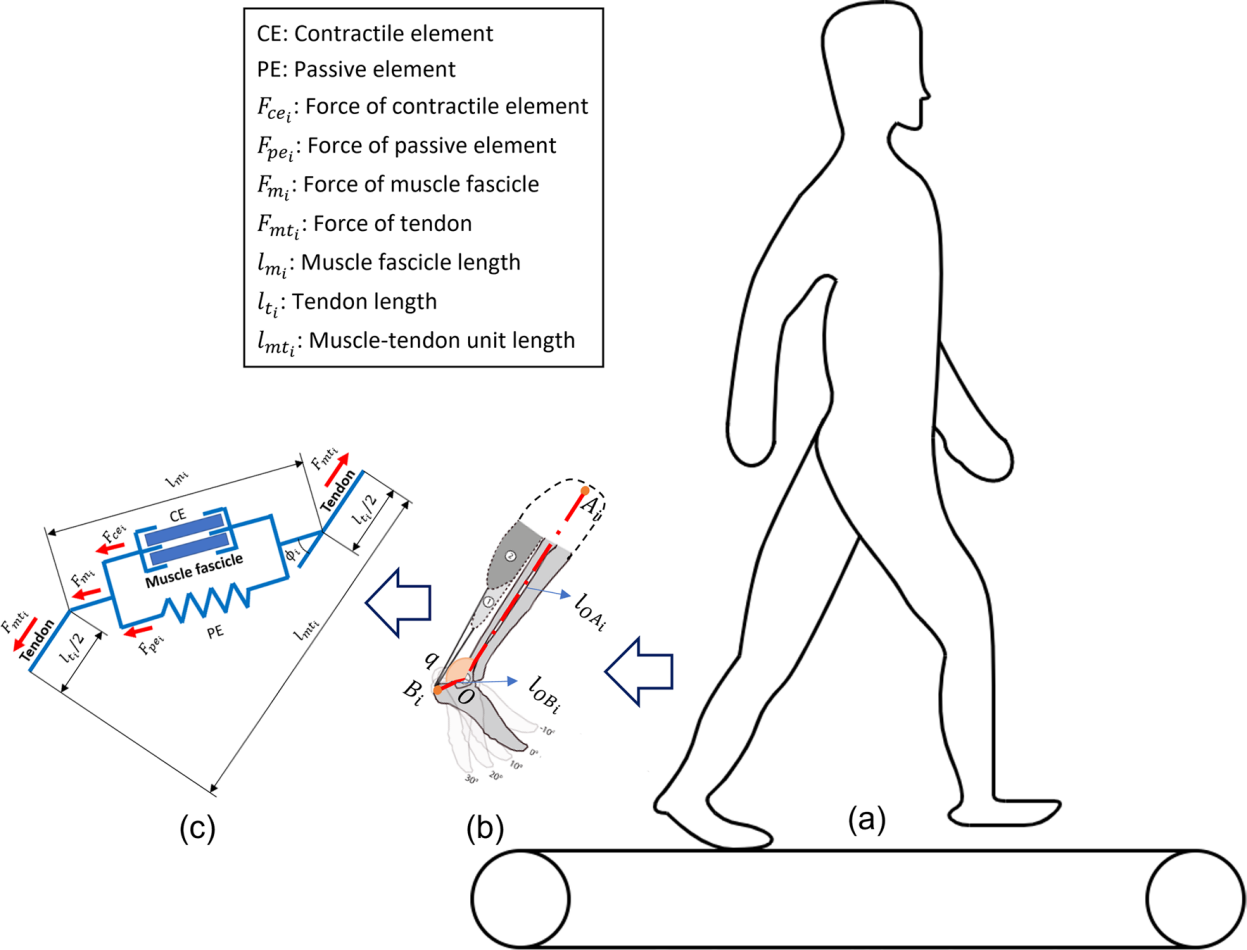
Schematic diagram of the muscle-tendon-unit (MTU) geometry model and contraction dynamic model of the plantarflexor muscles during treadmill walking tasks. **a** A human participant walking on a treadmill. **b** The MTU geometry model, where the ankle joint rotation center in the sagittal plane is denoted as *O*, and the proximal and distal osteotendinous junction points near the knee joint and heel are denoted as *A* and *B*. SOL and LGS muscles are referred as $i=1,2$. **c** The MTU contraction dynamic model, where the anatomical structure and contraction force generation of the MTU are illustrated

As presented in Fig. 1b, consider the ankle joint rotation center in the sagittal plane as *O*, the proximal and distal osteotendinous junction points of each each muscle-tendon unit (MTU) near the knee joint and heel as $A_{i}$ and $B_{i}$, and the angle between $OA_{i}$ and $OB_{i}$ as $q(t_{k})$, then each MTU length, $l_{mt_{i}}(t_{k})$, is represented as the distance between $A_{i}$ and $B_{i}$, and is calculated as

6 $$\begin{aligned} l_{mt_{i}}=\sqrt{l_{OA_{i}}^{2}+l_{OB_{i}}^{2}-2l_{OA_{i}}l_{OB_{i}}\cos (q)} \end{aligned}$$

where $l_{OA_{i}}$ and $l_{OB_{i}}$ represent the distance of $OA_{i}$ and $OB_{i}$ obtained from OpenSim (National Institutes of Health for Biomedical Computation, Stanford, USA) [45], respectively. A generic OpenSim model (*gait*2392) was linearly scaled to each participant in OpenSim version 4.1 [45] per [46]. According to the law of sines, the moment arm of each MTU, $r_{mt_{i}}(t_{k})$, is calculated as

7 $$\begin{aligned} r_{mt_{i}}=\frac{\partial l_{mt_{i}}(q)}{\partial (q)}=\frac{2l_{OA_{i}}l_{OB_{i}}\sin (q)}{l_{mt_{i}}}. \end{aligned}$$

The MTU generates contraction force only when it is stretched, which indicates the current tendon length $l_{t_{i}}(t_{k})$ is equal to or longer than the tendon slack length $l_{t_{i}}^{sk}$. From the perspective of muscle contraction dynamics that is shown in Fig. 1c, the overall MTU length, $l_{mt_{i}}(t_{k})$, could also be expressed as

8 $$\begin{aligned} l_{mt_{i}}=l_{t_{i}}+l_{m_{i}}\cos (\phi _{i}) \end{aligned}$$

where $l_{t_{i}}(t_{k})$ and $l_{m_{i}}(t_{k})$ represent the current tendon length and muscle fascicle length for LGS and SOL muscles, respectively. $\phi _{i}(t_{k})$ represents the pennation angle that changes with instantaneous $l_{m_{i}}(t_{k})$. By assuming the individual muscle belly has a relatively small MT change and volume change [47, 48], $\phi _{i}(t_{k})$ can be approximately calculated as

9 $$\begin{aligned} \phi _{i}\approx \arcsin \left( \frac{l_{m_{i}}^{0}\sin \phi _{i}^{0}}{l_{m_{i}}}\right) \end{aligned}$$

where the term $\phi _{i}^{0}$ denotes the constant pennation angle when the muscle is at optimal fascicle length $l_{m_{i}}^{0}$. The work in [49, 50] has shown that the optimal fascicle length increases as muscle activation decreases. Due to this coupling, the following relationship between the muscle activation and corresponding optimal fascicle length is given as

10 $$\begin{aligned} l_{m_{i}}^{0}=l_{mo_{i}}^{0}(\lambda (1-a_{i})+1) \end{aligned}$$

where $\lambda$ is the rate of change in the optimal fascicle length, and it is selected as 0.15 [39]. $l_{mo_{i}}^{0}$ is the optimal fascicle length at the walking task-specific maximum voluntary contraction, and $l_{m_{i}}^{0}(t_{k})$ is the optimal fascicle length at time $t_{k}$ and muscle activation $a_{i}(t_{k})$.

From the preliminary results of plantarflexors’ US imaging in [44], it is very challenging to capture the entire fascicle length $l_{m_{i}}(t_{k})$ of LGS or SOL muscles by using the current US transducer with a small dimension (width of 38 mm). Therefore, the fascicle length parameters of either LGS or SOL muscles were not measured directly from the US images. Instead, an indirect calculation method was applied as detailed below. According to [51], the nonlinear nominal tendon force-tendon strain relationship is given as

11 $$\begin{aligned} F_{t_{i}}(\xi _{i})=\left\{ \begin{array}{ll} 0, &{} \xi \le 0\\ 1480.3F_{i}^{\max }\xi _{i}^{2}, &{} 0<\xi <0.0127\\ (37.5\xi _{i}-0.2375)F_{i}^{\max }, &{} \xi \ge 0.0127 \end{array}\right. \end{aligned}$$

where $\xi _{i}=\frac{l_{t_{i}}(t_{k})-l_{t_{i}}^{sk}}{l_{t_{i}}^{sk}}$ represents the tendon strain and $F_{i}^{\max }$ represents the muscle contraction force at the walking task-specific maximum voluntary contraction. By considering $F_{t_{i}}(\xi _{i})=F_{mt_{i}}$, where $F_{mt_{i}}$ is defined in (13), $l_{t_{i}}(t_{k})$ can be numerically computed based on the Runga–Kutta–Fehlberg algorithm. By substituting (6) and (9) to (8), the muscle fascicle length $l_{m_{i}}(t_{k})$ will be calculated, as well as the muscle fascicle velocity $v_{m_{i}}(t_{k})$ by taking the time derivative of $l_{m_{i}}(t_{k})$.

#### Muscle dynamic contraction model

In the sEMG-US imaging-driven HNM, the individual moment component produced by each muscle, $M_{i}(t_{k})$, can be represented as

12 $$\begin{aligned} M_{i}=F_{mt_{i}}r_{mt_{i}} \end{aligned}$$

where $r_{mt_{i}}(t_{k})$ is defined in the above geometry model and $F_{mt_{i}}(t_{k})$ denotes the corresponding contraction force generated on each MTU and is represented as

13 $$\begin{aligned} F_{mt_{i}}=(F_{ce_{i}}+F_{pe_{i}})\cos (\phi _{i}) \end{aligned}$$

where $\phi _{i}(t_{k})$ represents the pennation angle defined above. $F_{ce_{i}}(t_{k})$ and $F_{pe_{i}}(t_{k})$ denote the corresponded forces generated by the parallelly located contractile element and the passive element, and can be calculated as

14 $$\begin{aligned} \begin{array}{lll} F_{ce_{i}} &{} = &{} F_{i}^{\max }f_{l_{i}}(l_{m_{i}})f_{v_{i}}(v_{m_{i}})a_{i}\\ F_{pe_{i}} &{} = &{} F_{i}^{\max }f_{p_{i}}(l_{m_{i}}) \end{array}. \end{aligned}$$

In (14), $F_{i}^{\max }$ of the LGS and SOL muscles will be identified based on optimization algorithm in the HNM calibration procedures. $f_{l_{i}}(l_{m_{i}}(t_{k}))$, $f_{v_{i}}(v_{m_{i}}(t_{k}))$, and $f_{p_{i}}(l_{m_{i}}(t_{k}))$ denote the generic muscle contractile force-fascicle length, force-fascicle velocity, and passive elastic force-fascicle velocity curves. These curves were normalized to $F_{i}^{\max }$, optimal fascicle length $l_{m_{i}}^{0}$, and maximum fascicle contraction velocity $v_{m_{i}}^{\max }$. The explicit expressions of $f_{l_{i}}(l_{m_{i}}(t_{k}))$, $f_{v_{i}}(v_{m_{i}}(t_{k}))$, and $f_{p_{i}}(l_{m_{i}}(t_{k}))$ can be found in [25, 32, 52].

### Experimental protocol, data collection and pre-processing

**Fig. 2:**
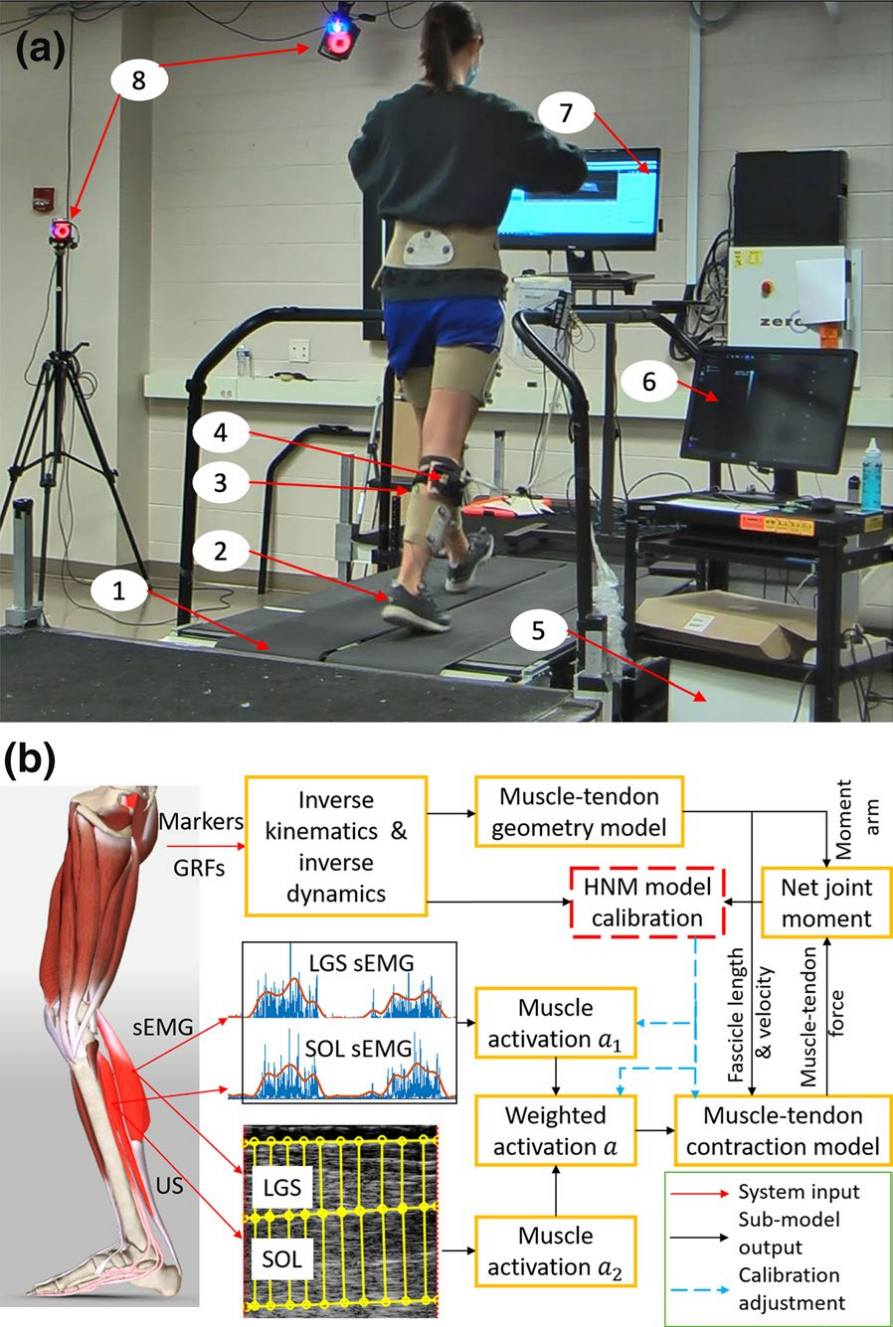
The treadmill walking experimental setup, data collection, processing, and Hill-type neuromuscular model calibration. **a** Illustration of walking experimental setup. (1) Instrumented treadmill with two split belts and in-ground force plates. (2) 39 retro-reflective markers on participant’s lower body for lower limb kinematics measurements. (3) Four sEMG sensors to record signals from LGS, MGS, SOL, and tibialis anterior (TA) muscles. (4) A single US transducer to image both LGS and SOL muscles with the appropriate probe placement. (5) Ultrasound imaging machine to collect radio frequency data. (6) Computer screen to show B-mode ultrasound imaging. (7) Computer screen to show live markers and segment links of the participant. (8) 12 motion capture cameras to track markers’ trajectories. **b** Schematic illustration of the proposed sEMG-US imaging-driven HNM calibration. The weighted muscle activation for LGS or SOL combines both sEMG signals and US imaging. Red solid, black solid, and blue dashed lines with an arrow represent the input signals to HNMs, the intermediate sub-model output signals, and the unknown parameters adjustment, respectively

The study was approved by the Institutional Review Board (IRB) at North Carolina State University (IRB number: 20602). Ten young participants (7M/3F, age: 25.4 ± 3.1 years, height: 1.77 ± 0.10 m, mass: 78.0 ± 21.1 kg) without any neuromuscular or orthopedic disorders, were recruited in this study. Every participant got familiar with the experimental procedures and signed an informed consent form before participation.

Figure 2a summarizes the experimental setup for the participants to perform static anatomical poses and dynamic gait trials (walking speeds changing from 0.50 to 1.50 m/s), and Fig. 2b presents the workflow of the data processing and sEMG-US imaging-driven HNM calibration procedures. During all walking trials, three-dimensional coordinates of 39 retro-reflective markers positioned on the participant’s lower extremities following the instructions in [53] were recorded using a 12-camera motion capture system (Vicon Motion Systems Ltd, Los Angeles, CA, USA) at 100 Hz. The GRF signals were collected at 1000 Hz synchronously with makers trajectories using in-ground force plates (AMTI, Watertown, MA, USA) mounted on an instrumented treadmill (Bertec Corp., Columbus, OH, USA) through the commercial real-time data capture software Nexus 2.9. Both GRF signals and markers trajectories were low-pass filtered with a fourth-order Butterworth filter in Visual 3D software (C-Motion, Rockville, MD, USA), and the cut-off frequencies were set as 25 Hz and 6 Hz, respectively. The markers trajectories and GRF signals from static poses and dynamic gait trials were used for joints kinematics and net joint moment calculation by using inverse kinematics and ID algorithms in Visual 3D. During all trials, four wired sEMG sensors (SX230, Biometrics Ltd, Newport, UK) were attached to the shank skin through a double-sided tape to non-invasively record the electrical signals from tibialis anterior (TA), LGS, MGS, and SOL muscles at 1000 Hz synchronously through Nexus 2.9. The locations for sEMG sensors were determined following the instructions in [54]. A US transducer (38-mm of length, 6.4 MHz center frequency, L7.5SC Prodigy Probe, S-Sharp, Taiwan) setup was applied in the walking experiments as described in [34]. The US radio frequency (RF) data were recorded at 1000 frames per second synchronously with markers trajectories, GRF, and sEMG signals, using a pulse sequence trigger signal generated from Nexus 2.9.

sEMG signals were band-pass filtered with the bandwidth between 20 Hz and 450 Hz, and then full-wave rectified and low-pass filtered with a cut-off frequency of 6 Hz. The resulting linear envelopes were normalized to the peak processed sEMG values obtained from all sets of dynamic gait trials with different speeds. The US radio frequency data were converted to B-mode images through beamforming and logarithmic compression, and then a commercial US imaging processing toolbox *UltraTrack* [55] was applied to determine the temporal sequences of the LGS and SOL muscles’ thickness based on the adaptive optical flow tracking algorithms. First, one region of interest (width by height: 336 $\times$ 400 pixels) that encompassed the LGS and SOL muscles was selected as the area between the superficial and deep aponeuroses. Then, 10 vertical lines were manually defined with evenly distributed distances in the LGS’s region and SOL’s region on the first US imaging frame for each recorded waking trial, as shown in Fig. 2b. Key-frame correction [55] was applied to minimize the time-related drift of MT’s cyclical pattern over multiple gait cycles, where the key frames were selected to be at heel-strike and toe-off time points. After each correction, the new key frames’ vertical lines positions were determined by applying an affine transformation to the key-frame before it. Finally, the temporal sequence of the mean value of those 10 vertical lines’ lengths from LGS and SOL muscles were calculated for LGS’s MT and SOL’s MT, respectively. The MT signals were then low-pass filtered with a cut-off frequency of 30 Hz.

Before data collection, participants practiced dynamic walking steadily with all sensing devices attached to the lower extremities on the treadmill for at least 30 s for each speed. The duration of each walking trial was set as 2 min, and data from the middle 20 s were collected for analysis. In the current study, there were five different walking speeds, 0.50 m/s, 0.75 m/s, 1.00 m/s, 1.25 m/s, and 1.50 m/s, and the order was selected randomly for each participant. The participants were provided with at least 2 min for rest between two successive dynamic walking trials to avoid muscle fatigue.

### Data post-processing

#### HNM model calibration

To determine the values for a set of parameters, including the shape factor $A_{i}$, the tendon slack length $l_{t_{i}}^{sk}$, the muscle contraction force at the walking task-specific maximum voluntary contraction $F_{i}^{\max }$, and the muscle activation allocation gain $\delta _{i}$, the HNM model calibration was performed with the initial parameters obtained from the literature [56]. The summarized calibration process of the sEMG-US imaging-driven HNM is presented in Fig. 2b. Indeed, the model calibration is a nonlinear optimization problem, where the above parameters set needs to be found for minimizing the designated objective function. During calibration, the parameters were subsequently adjusted within the predefined boundaries, which ensured the muscle-tendon unit always operated within the physiological range [39]. Except for the allocation gains $\delta _{i}$ were set the range between 0 and 1, other parameters were set with the lower and upper bounds as 50% and 150% of the literature-referred values, while the initial guess of the set of parameters were set as the middle value of each parameter. Other HNM parameters, like muscle-tendon unit length $l_{mt_{i}}$, optimal muscle fascicle length $l_{mo_{i}}^{0}$, and optimal pennation angle $\phi _{i}^{0}$ were assigned using OpenSim and the scaling method as references. The Matlab function ’lsqcurvefit’ with the Levenberg-Marquardt algorithm was applied to solve the nonlinear least-squares optimization problem until the following objective function was minimized during the calibration

15 $$\begin{aligned} E_{obj}=\frac{1}{N}\sum _{j=1}^{N}\left( \left( \sum _{i=1}^{2}M_{i}(j)\right) -M_{w}(j)\right) ^{2} \end{aligned}$$

where $\sum _{i=1}^{2}M_{i}(j)$ represents the net PF moment estimation from both LGS and SOL muscles during stance phase at time instant *j*, $M_{w}(j)$ represents the net PF moment measurement from ID at time instant *j*, and *N* represents the length of the data used for the calibration.

It should be noted that the HNM calibration is subjective, and for each participant, the calibration procedure was repeated for both single-speed modes and inter-speed mode. In the current study, single-speed modes are defined as the HNM calibration only with data collected from one single speed (five single-speed modes here), while the inter-speed mode is defined as the HNM calibration with all data collected from five different speeds (one inter-speed mode here). In HNM calibration, collected data, including ankle joint’s kinematics, kinetics, sEMG signals, and US imaging from the stance phase of 10 steady gait cycles, were used to minimize the objective function in (15). For the single-speed modes, the 10 steady gait cycles were all from one speed, while for the inter-speed mode, the 10 gait cycles were composed of two steady cycles from each of five speeds. Similarly, by manually setting the muscle activation allocation gain $\delta _{i}$ to be 1 and 0, we could also conduct the calibration procedures of the sEMG-driven and US imaging-driven HNMs. After the HNM calibration with different modes, new data sets (five gait cycles not involved in the calibration procedures) from walking trials at different speeds were used as input of the calibrated HNMs to predict net PF moment. The prediction results were evaluated through the comparison to the measured net PF moment from ID.

#### Statistical analysis

The validation procedures comprised three tests to assess the sEMG-US imaging-driven, sEMG-driven, and US imaging-driven HNMs’ calibration and prediction abilities of the net ankle joint PF moment during the stance phase. In each type of HNM test, the root mean square error (*RMSE*), *RMSE* normalized to individual peak net PF moment ($N-RMSE$), *RMSE* normalized to individual body mass ($BM-RMSE$), and coefficient of determination ($R^{2}$) values between the calibrated/predicted net PF moment and ID-calculated net PF moment were calculated to evaluate the calibration/prediction performance. The rational of using these three tests is given here. From a mathematical perspective, the proposed sEMG-US imaging-driven HNM is a model-based regression problem. Given that the corresponding data were all collected continuously and synchronously, and the ground truth from the ID calculation and the prediction from different HNMs are all continuous, the most intuitive metric to evaluate the prediction accuracy is to use the *RMSE* between the ground truth and prediction. However, due to the weight, height, and walking pattern’s variations among subjects, the capability (peak value) of net plantarflexion moment generation during treadmill walking at the same speed is different, which would affect the prediction *RMSE* directly. Therefore, the direct *RMSE* values comparison among subjects is likely to introduce high deviations. An effective way to reduce the subjective variation of the prediction *RMSE* is to take the normalization to a subjective characteristic, like the body mass and peak net plantarflexion moment as we selected. The relationship between $N-RMSE$ and $BM-RMSE$ is that in the normalization calculation, they have same numerators but different denominators for the same subject. Although $N-RMSE$ and $BM-RMSE$ might provide potential redundancy information as can be seen from subsequent calibration and prediction summary results, they show different sensitivities to the walking speeds ($BM-RMSE$ is more sensitive). Another useful metric to evaluate the prediction accuracy is the coefficient of determination, known as $R^{2}$, which ranges from 0 to 1 and measures the proportion of variation in the data that is accounted for in the model. In the current study, $R^{2}$ is used as a parallel evaluation metric as N-RMSE and BM-RMSE, and it is relatively not sensitive to the walking speeds. Although $R^{2}$ and $N-RMSE$ might provide potential redundancy information, it is not always true that lower $N-RMSE$ corresponds to higher $R^{2}$, so we keep both to provide potential supplementary information.

Shapiro-Wilk parametric hypothesis test was used to determine the normality of the corresponding $N-RMSE$, $BM-RMSE$, and $R^{2}$ values of each calibration step under the single-speed modes and inter-speed mode, and each prediction step. According to the results of the Shapiro-Wilk test, two-way repeated-measure analysis of variance (ANOVA) or Friedman’s tests followed by a Tukey’s honestly significant difference tests (Tukey’s HSD) was applied to evaluate the effect of HNMs’ category and walking speed on different evaluation criteria, including $N-RMSE$, $BM-RMSE$, and $R^{2}$ values, during both calibration and prediction procedures. The significant difference level was chosen as $p<0.05$ for all statistical tests. Effect sizes were reported as $\eta _{p}^{2}$ and Cohen’s *d* for main effects from ANOVA or Friedman’s tests and pairwise comparisons from Tukey’s HSD, respectively.

## Results

### Ankle joint kinematics and kinetics, and plantarflexors neuromuscular features

**Fig. 3:**
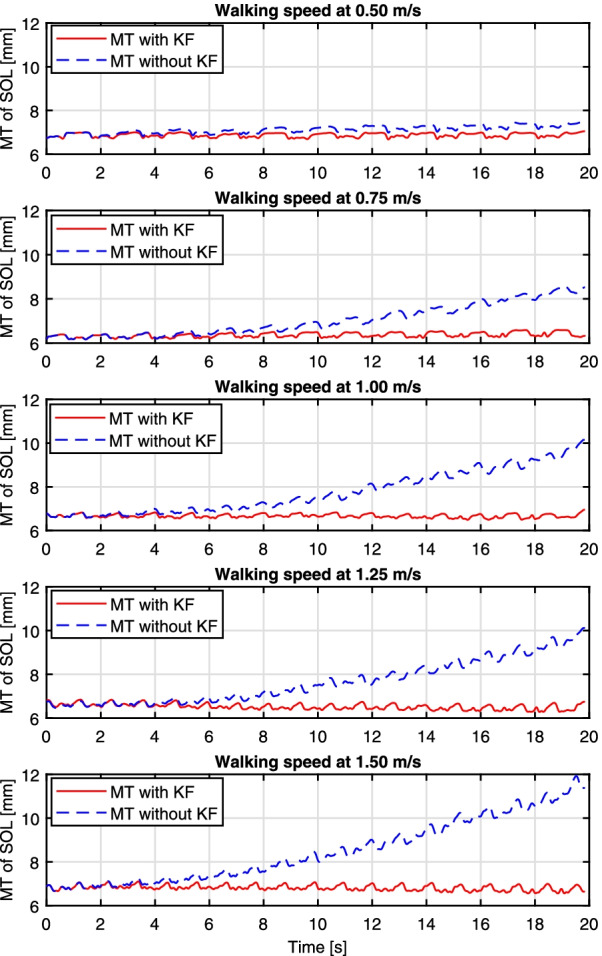
SOL muscle thickness tracking results by using *UltraTrack* at different walking speeds on Participant Sub08. Each subplot represents the tracking results during the recorded 20 s experiments under each walking speed, and the red solid and blue dashed lines represent the results with and without key-frame correction, respectively

The treadmill walking speed’s effect on human ankle joint kinematics, kinematics, and lower extremities’ muscles activities has been extensively investigated and discussed in a recent study [57], but without results related to the architectural change of those muscles. In this study, the time sequences of both LGS and SOL’s muscle thickness during the recorded walking duration were extracted by using *UltraTrack*. Take the SOL muscle of Participant Sub08 as an example, Fig. 3 shows the MT tracking results throughout the 20 s walking duration at each walking speed. The red solid and blue dashed curves represent the MT change of the SOL muscle with and without key-frame correction, respectively. The results demonstrate that the key-frame correction could significantly reduce the time-related drift, which is due to the tracking error accumulation along with the walking duration (exampled US imaging video with the LGS and SOL’s MT tracking results at multiple walking speeds can be referred to Additional files 2, 3, 4, 5, 6). The reported results here are consistent with the studies mentioned in [55].

**Fig. 4:**
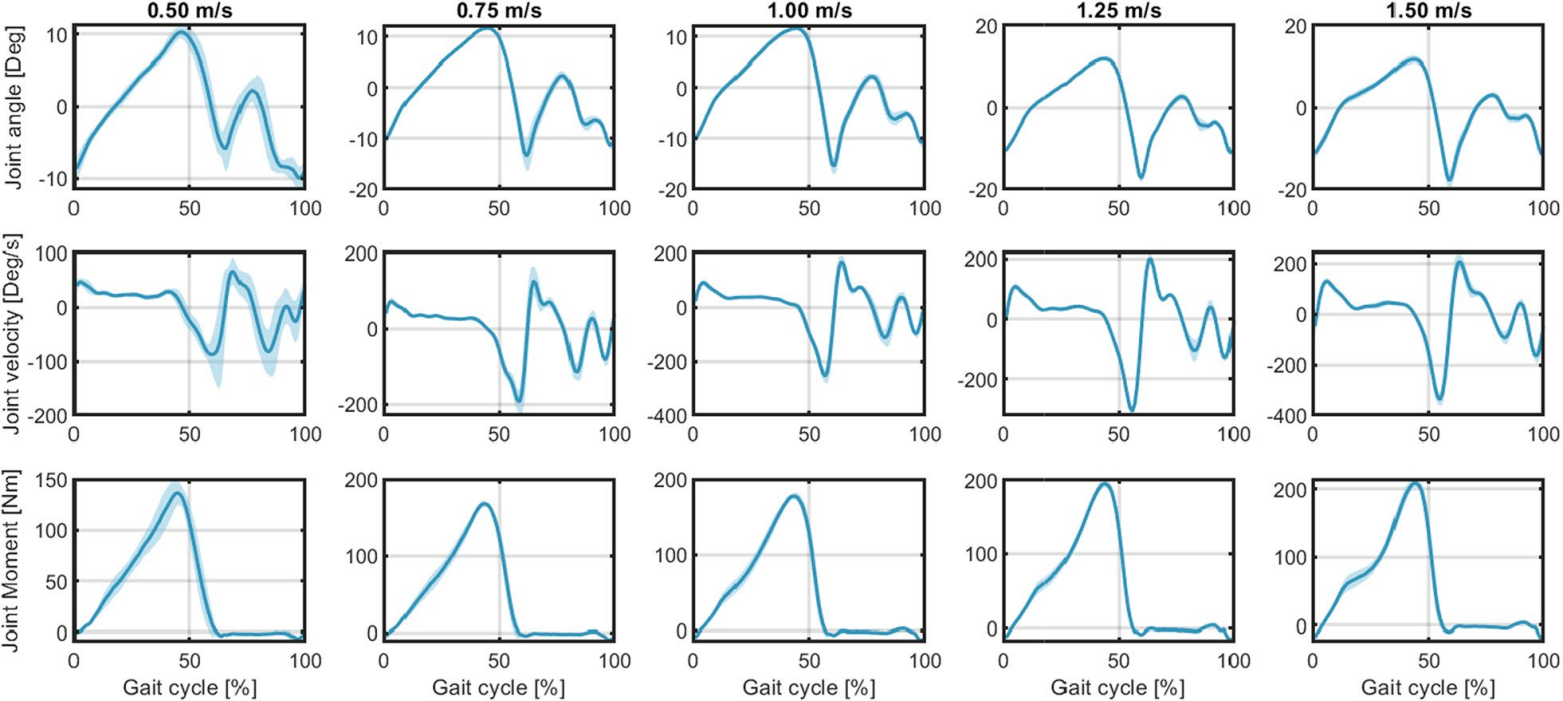
Results of joint kinematics and kinetics during the treadmill walking experiments at different speeds on Participant Sub01. The mean and one SD values on the right ankle joint during recorded gait cycles (between the heel-strike instants in the current and the consecutive gait cycles) are represented by the solid lines and the light shadowed areas, respectively

**Fig. 5:**
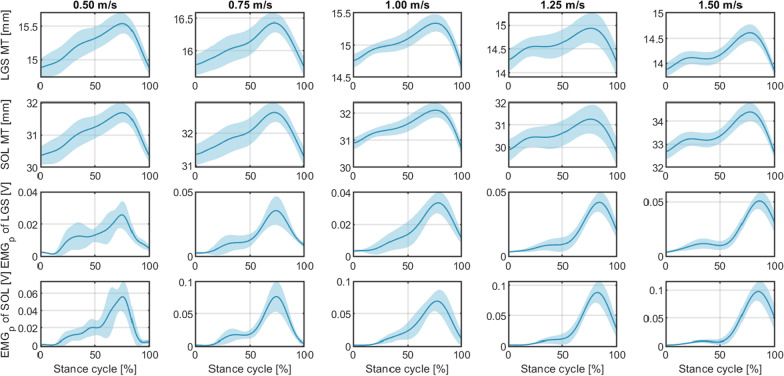
Results of processed sEMG signals and US imaging-derived MT signals from both LGS and SOL muscles during the treadmill walking experiments at different speeds on Participant Sub01. The mean and one SD values of the plantarflexor muscles on the right shank during recorded stance phase cycles (from the heel-strike instant to the toe-off instant in the same gait cycle) are represented by the solid lines and the light shadowed areas, respectively

The continuous results of the ankle joint kinematics and kinetics throughout the recorded 20 seconds at each speed were segmented as a percent of gait cycle from 0 to 100% according to the GRF measurements, which are shown in Fig. 4. The solid lines and light shadowed areas report the mean and one standard deviation (SD) values of the right ankle joint angular position, velocity, and net PF moment changes across all gait cycles at each walking speed on Sub01 (data from other participants can be found in the Additional file 1: Fig. S1–S5), where 0% and 100% represent the time instants when the heel-strike occurred in the current and consecutive gait cycles, respectively. By comparing the ankle joint moment calculated from ID, the averaged peak net PF moment across all walking gait cycles within each walking trial monotonically increased with the increase of walking speeds on each participant, indicating more power was needed for faster walking speed. The continuous results of neuromuscular features throughout the recorded walking duration were segmented as a percent of stance cycle from 0 to 100%, which are shown in Fig. 5. The solid lines and light shadowed areas report the mean and one SD values of the processed sEMG signals and US imaging-derived MT signals from the LGS and SOL muscles on the right leg during multiple stance phase cycles at each walking speed on Sub01 (data from other participants can be found in the Additional file 1: Fig. S11–S15), where 0% and 100% represent the time instants when the heel-strike and toe-off occurred in the same gait cycle, respectively. The upper two rows present the LGS and SOL MT changes during the stance phase, while the lower two rows present the muscle’s low-pass filtered sEMG changes. The results show small variations of MT and sEMG for both LGS and SOL muscles at heel-strike across all walking speeds, but relatively high variations of sEMG for both LGS and SOL muscles at toe-off, where the sEMG signals increase with increased walking speed. For all five walking speeds, we observe that MT of LGS or SOL muscle is almost identical at heel-strike and toe-off, however, the processed sEMG signals of LGS or SOL muscle show higher value at toe-off than at heel-strike. By comparing the shadowed areas of the same feature across speeds, we observed that features’ deviations are higher at a slower speed, like 0.50 m/s, than those at higher speeds. This implies that keeping a steady muscle contraction pattern at a slower speed is more difficult than at a higher speed.

### Correlation analysis results

Overall, in Fig. 5, both temporal MT and sEMG signals from LGS and SOL muscles show a fairly strong correlation with the net ankle joint PF moment calculated from ID. For each participant, it was assumed the step-to-step variation is negligible, so we segmented all periods of the ankle joint net plantarflexion moment during the walking stance phase at each walking speed, and then calculated a correlation coefficient value between the net plantarflexion moment time sequence and each neuromuscular feature time sequence during the same stance cycle. Therefore, for a specific neuromuscular feature and walking speed, if the stance cycles are $n_{1}, n_{2}, ..., n_{10}$ for all ten participants, the number of correlation coefficients would be $\sum _{i=1}^{10}n_{i}$. To address the person-to-person variation and to get each entry in Table 1, we applied the Fisher transformation to yield approximately normally distributed correlation coefficients. After the Fisher transformation, we calculated the mean and one SD values of the correlation coefficients for each walking speed and the neuromuscular feature, as listed in Table 1. The results show that a positive relationship exists between the processed LGS or SOL’s sEMG signal and net PF moment, and between the LGS or SOL’s MT and net PF moment. All the mean values of correlation coefficients are higher than 0.8 except for the SOL’s sEMG signal at 1.50 m/s walking speed. From the results of a two-way repeated-measure ANOVA, we did not observe a significant difference in the correlation coefficient values among the four neuromuscular features ($p=0.201$) or among the walking speed ($p=0.112$), which indicates the MT and sEMG signals might have comparable capabilities to predict net PF moment across speeds.

**Table 1:** Correlation coefficients (mean ± SD) between each neuromuscular feature and the net PF moment across all stance phase cycles and ten participants at each walking speed

	Neuromuscular features			
	LGS sEMG	SOL sEMG	LGS MT	SOL MT
*Speed [m/s]*				
0.50	0.886 (0.019)	0.876 (0.086)	0.857 (0.052)	0.867 (0.053)
0.75	0.890 (0.058)	0.905 (0.054)	0.842 (0.024)	0.865 (0.032)
1.00	0.892 (0.066)	0.896 (0.056)	0.883 (0.063)	0.892 (0.056)
1.25	0.835 (0.096)	0.857 (0.076)	0.840 (0.034)	0.833 (0.033)
1.50	0.801 (0.124)	0.785 (0.081)	0.847 (0.018)	0.844 (0.022)

### Results of HNM calibration and net PF moment prediction

**Fig. 6:**
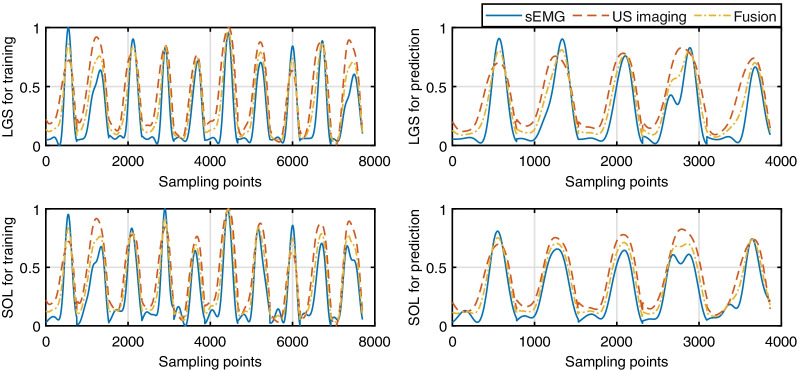
Muscle activation levels of both LGS and SOL during the walking stance phase by setting the allocation gain as 0, 1, and the optimal value, respectively. The blue solid, red dashed, and orange centered curves represent the muscle activation levels by using only sEMG signals, only US imaging-derived MT signals, and the fusion between sEMG and MT signals with the optimal allocation gain in both model calibration (left column) and prediction (right column) procedures. Data shown in the figure are from Participant Sub03 when walking on the treadmill at 0.75 m/s

**Fig. 7:**
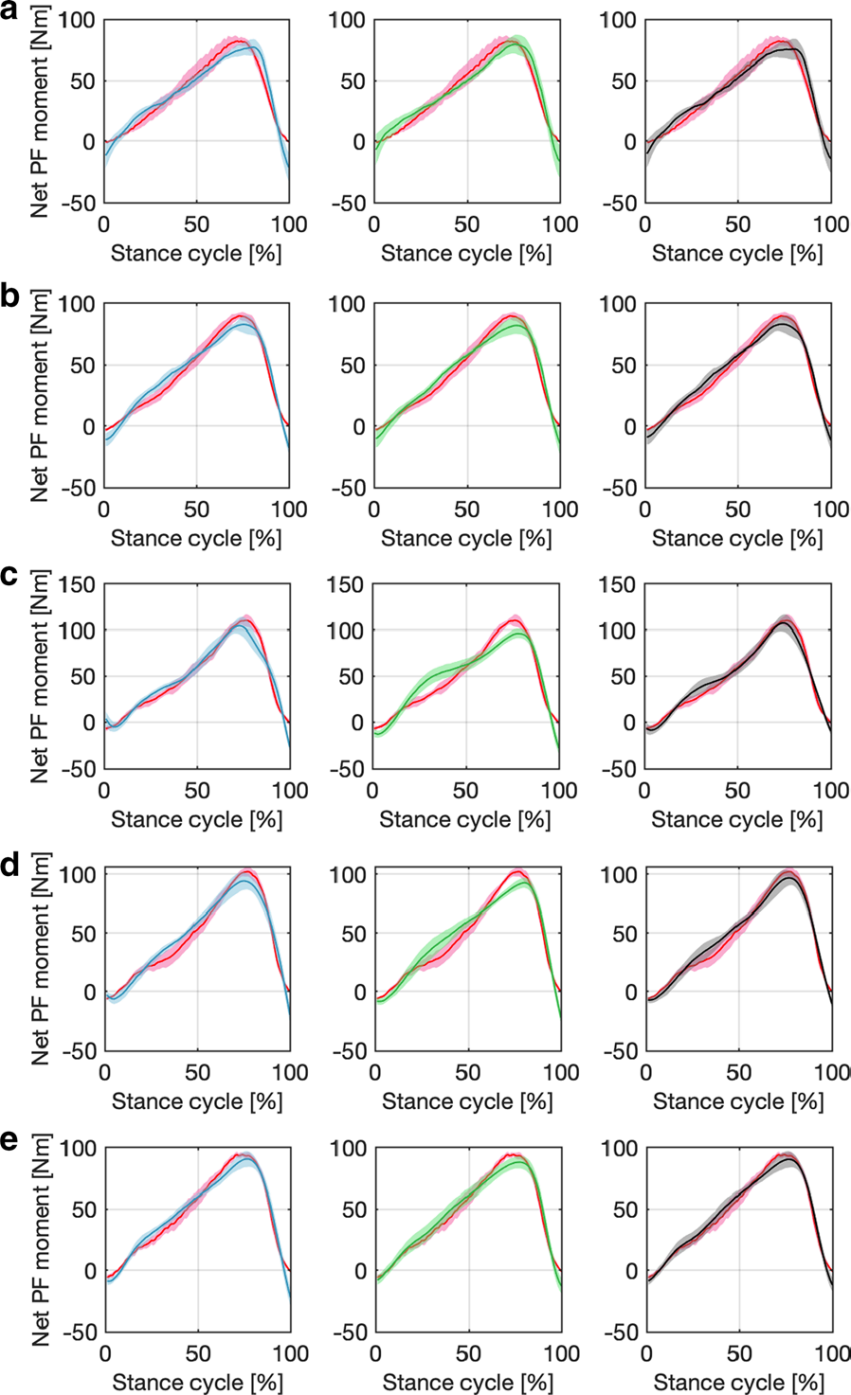
Calibration results by using three HNM categories on Participant Sub05 under single-speed modes. The calibration performance by using sEMG-, US imaging-, and sEMG-US imaging-driven HNMs under 5 single-speed modes on Sub05. The red lines and light shadowed areas represent the mean and SD of the net PF moment from the ID calculation, the blue, green, black lines and light shadowed areas represent the mean and SD of the net PF moment from calibrations by using sEMG-driven HNM, US imaging-driven HNM, and sEMG-US imaging-driven HNM, respectively. **a–e** represent the results at each walking speed from 0.50 to 1.50 m/s

In the HNM calibration procedures shown in Fig. 2b, one essential input component is the muscle activation levels of both LGS and SOL muscles, either only based on sEMG signals, US imaging-derived MT signals, or data fusion between them. The representative demonstration of three types of muscle activation levels of both LGS and SOL muscles during the walking stance phase at 0.75 m/s on Participant Sub03 are shown in Fig. 6. The left and right column subplots represent the time sequence points of muscle activation levels that are used in the model calibration and prediction procedures. It is observed that the data fusion could balance the muscle activation levels from both sEMG signals and MT signals and further compensate for the activation level drift from only MT signals, which is potentially beneficial for the accuracy improvement of the net plantarflexion moment prediction. Fig. 7 presents the representative results of HNMs calibrations on Sub05 with data collected from each individual walking speed, where each solid centered line and the shadowed area represent the mean and one SD values of the measured and calibrated net ankle joint PF moment, and each curve is composed of 10 stance cycles at each walking speed. For this participant, the mean calibration *RMSE* values are 8.70 N$\cdot$m, 9.10 N$\cdot$m, and 7.90 N$\cdot$m by using sEMG-, US imaging-, and sEMG-US imaging-driven HNMs under walking speed of 0.50 m/s, respectively. Results from other walking speeds also showed that the sEMG-US imaging-driven HNM could effectively reduce the calibration *RMSE* under either single-speed modes or inter-speed mode. To quantitatively evaluate the calibration performance by using different HNM categories and under different speed modes, the mean *RMSE* and $R^{2}$ values between the benchmark moment values from ID and calibrated moment values on each participant are summarized in Table 2. Based on the observation from Fig. 7, it appears that the relative error may be inflated during specific areas of the stance cycles. The current outcomes (i.e., $N-RMSE$, $BM-RMSE$, and $R^2$ values) may not be directly sensitive to those specific areas, because the three evaluation metrics report an averaged performance across the entire stance cycle. This limitation of the current outcome metrics can be addressed in future work by identifying systematic changes in the prediction accuracy throughout the stance phase cycle rather than across the entire cycle.

**Table 2:** Calibration results with three HNM categories in both single-speed modes and inter-speed mode on each participant

Subject	HNMs	Single-speed modes					
		0.50 m/s		0.75 m/s		1.00 m/s	
		*RMSE*	$R^{2}$	*RMSE*	$R^{2}$	*RMSE*	$R^{2}$
Sub01	sEMG	12.16	0.954	11.28	0.956	12.75	0.954
	US imaging	14.49	0.921	14.62	0.926	12.94	0.951
	sEMG-US imaging	11.79	0.965	10.25	0.964	11.77	0.960
Sub02	sEMG	5.96	0.932	6.93	0.936	7.70	0.934
	US imaging	5.91	0.932	6.44	0.943	7.07	0.945
	sEMG-US imaging	4.94	0.953	5.10	0.964	6.91	0.947
Sub03	sEMG	9.86	0.924	8.38	0.964	11.28	0.949
	US imaging	13.68	0.857	11.17	0.937	12.29	0.940
	sEMG-US imaging	8.33	0.946	6.49	0.979	9.81	0.962
Sub04	sEMG	12.04	0.820	17.14	0.842	13.79	0.904
	US imaging	16.60	0.661	20.49	0.771	16.38	0.865
	sEMG-US imaging	11.64	0.834	16.89	0.847	13.48	0.908
Sub05	sEMG	8.70	0.903	9.43	0.904	9.01	0.921
	US imaging	9.10	0.894	9.77	0.897	9.54	0.911
	sEMG-US imaging	7.90	0.920	8.96	0.913	8.97	0.921
Sub06	sEMG	6.36	0.963	6.15	0.974	9.35	0.953
	US imaging	7.08	0.954	12.42	0.897	18.34	0.823
	sEMG-US imaging	5.53	0.972	6.02	0.976	9.28	0.954
Sub07	sEMG	5.76	0.923	6.75	0.921	6.84	0.949
	US imaging	6.22	0.911	8.34	0.879	7.31	0.942
	sEMG-US imaging	5.46	0.932	6.48	0.927	6.67	0.951
Sub08	sEMG	4.98	0.973	6.64	0.957	9.20	0.936
	US imaging	4.97	0.973	6.97	0.953	8.34	0.946
	sEMG-US imaging	4.61	0.977	6.35	0.961	8.24	0.948
Sub09	sEMG	7.37	0.926	8.65	0.919	7.76	0.941
	US imaging	8.99	0.894	9.55	0.901	8.13	0.936
	sEMG-US imaging	7.22	0.929	8.27	0.926	7.24	0.949
Sub10	sEMG	9.31	0.935	7.27	0.973	12.24	0.940
	US imaging	13.85	0.856	11.97	0.927	12.56	0.937
	sEMG-US imaging	8.25	0.949	5.78	0.983	9.72	0.962

Subject	HNMs	Single-speed modes				Inter-speed mode	
		1.25 m/s		1.50 m/s		All speeds	
		*RMSE*	$R^{2}$	*RMSE*	$R^{2}$	*RMSE*	$R^{2}$
Sub01	sEMG	13.40	0.955	14.59	0.953	14.42	0.936
	US imaging	13.85	0.952	13.38	0.960	18.79	0.893
	sEMG-US imaging	12.61	0.960	12.76	0.964	12.95	0.949
Sub02	sEMG	13.47	0.823	11.29	0.874	10.62	0.862
	US imaging	16.86	0.724	13.76	0.816	12.28	0.818
	sEMG-US imaging	13.41	0.822	8.79	0.922	10.01	0.877
Sub03	sEMG	11.55	0.950	12.07	0.946	13.53	0.924
	US imaging	11.62	0.950	13.01	0.938	16.19	0.916
	sEMG-US imaging	9.38	0.967	11.30	0.952	11.38	0.951
Sub04	sEMG	14.91	0.893	14.89	0.913	17.14	0.848
	US imaging	17.26	0.857	15.13	0.911	23.52	0.723
	sEMG-US imaging	14.87	0.894	14.86	0.914	17.12	0.849
Sub05	sEMG	6.79	0.963	8.15	0.954	10.60	0.892
	US imaging	6.87	0.961	8.51	0.949	12.97	0.840
	sEMG-US imaging	6.67	0.964	8.08	0.954	10.48	0.895
Sub06	sEMG	8.74	0.964	18.06	0.884	13.13	0.905
	US imaging	13.54	0.915	15.99	0.908	17.32	0.832
	sEMG-US imaging	8.37	0.967	13.91	0.931	11.76	0.922
Sub07	sEMG	7.66	0.941	10.59	0.898	9.46	0.890
	US imaging	7.38	0.946	9.37	0.920	10.41	0.868
	sEMG-US imaging	7.04	0.951	9.02	0.927	8.64	0.909
Sub08	sEMG	11.74	0.912	14.98	0.864	14.98	0.864
	US imaging	10.82	0.925	14.27	0.877	14.27	0.877
	sEMG-US imaging	10.44	0.931	13.76	0.886	13.76	0.886
Sub09	sEMG	7.55	0.925	8.02	0.954	10.58	0.929
	US imaging	9.64	0.878	8.28	0.923	14.38	0.870
	sEMG-US imaging	6.87	0.955	7.92	0.955	10.08	0.933
Sub10	sEMG	12.10	0.946	10.85	0.956	13.14	0.939
	US imaging	12.89	0.939	11.86	0.948	17.26	0.906
	sEMG-US imaging	10.19	0.962	10.08	0.962	12.37	0.960

(*RMSE* unit: N$$\cdot$$m)

**Fig. 8:**
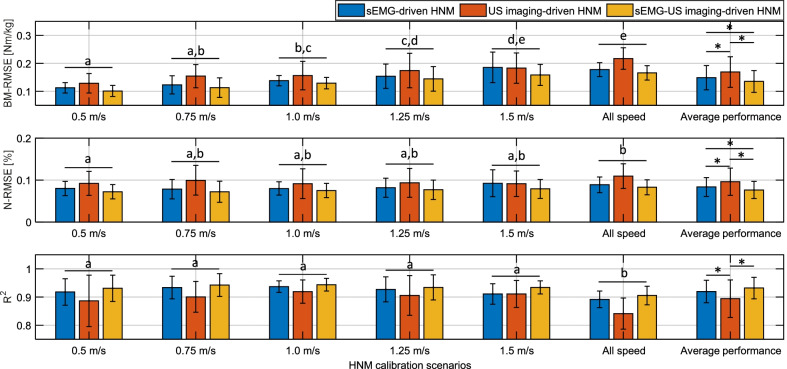
Calibration performance across all participants by using three HNM categories. The mean and SD of $BM-RMSE$, $N-RMSE$, and $R^{2}$ values across all participants under both single-speed modes and inter-speed mode are displayed in upper, middle, and lower plots, respectively, during calibration procedures. Blue, red, and yellow bars represent the results by using sEMG-, US imaging-, and sEMG-US imaging-driven HNMs, respectively. Letters and asterisks represent the statistically significant difference from the two-way repeated-measure ANOVA followed by a Tukey’s HSD, where letters stand for the evaluation of the walking speed factor (significant difference only exists between each two bar groups out of the left six groups without any overlap of letters) and asterisks stand for the evaluation of the applied HNM type factor (significant difference exists between each two bars out of the most right group when the asterisk is there)

Overall, the calibration *RMSE*/$R^{2}$ values by using the sEMG-US imaging-driven HNM are lower/higher than those by using the sEMG-driven and US imaging-driven HNMs, respectively, for both single-speed modes and inter-speed mode on all participants. Additionally, 97.5 % of $R^{2}$ values are higher than 0.80, which indicates a strong linear correlation between the calibrated and ID-calculated net PF moment values. After calculating the $N-RMSE$ and $BM-RMSE$ values, the summarized calibration performance across all participants under both single-speed modes and inter-speed mode is presented in Fig. 8. Shapiro-Wilk tests show that all $N-RMSE$, $BM-RMSE$, and $R^{2}$ values under the single-speed modes and inter-speed mode in both calibration and prediction are normally distributed. In calibration, results across participants from the two-way ANOVA show $BM-RMSE$ values systematically changed in response to changes in speed (main effect, $p<0.001$, $n_{p}^{2}=0.268$) and applied HNMs (main effect, $p<0.001$, $n_{p}^{2}=0.162$). $N-RMSE$ values systematically changed in response to changes in speed (main effect, $p<0.001$, $n_{p}^{2}=0.089$) and applied HNMs (main effect, $p<0.001$, $n_{p}^{2}=0.193$). $R^{2}$ values were significantly affected by speed (main effect, $p<0.001$, $n_{p}^{2}=0.153$) and applied HNMs (main effect, $p<0.001$, $n_{p}^{2}=0.186$). However, there is no evidence of an interaction effect of the speed and HNM on $BM-RMSE$ values ($p=0.679$), $N-RMSE$ values ($p=0.948$), and $R^{2}$ values ($p=0.279$), respectively. Additionally, the results from the post hoc Tukey’s HSD are also marked in Fig. 8, where letters and asterisks represent the statistically significant difference.

**Fig. 9:**
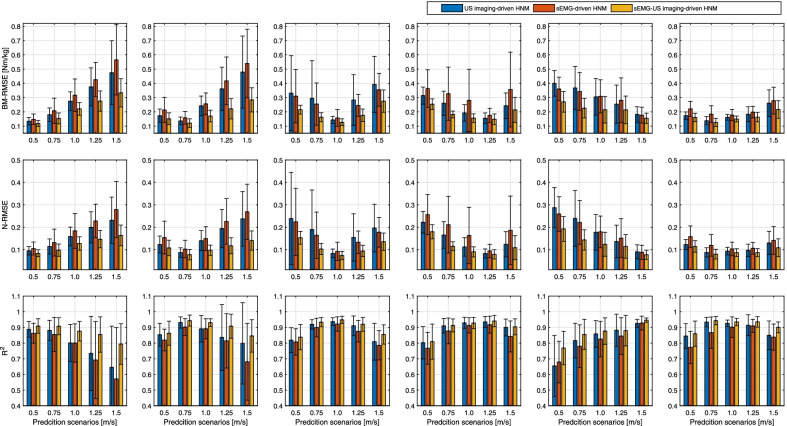
Prediction performance across all participants by using three HNM categories. The mean and SD of $BM-RMSE$, $N-RMSE$, and $R^{2}$ values across all participants under both single-speed modes and inter-speed model are displayed in upper, middle, and lower row plots, respectively. Blue, red, and yellow bars represent the prediction results by using sEMG-, US imaging-, and sEMG-US imaging-driven HNMs, respectively. Column subplots from left to right represent that these applied HNMs were calibrated under single-speed modes (0.50 m/s, 0.75 m/s, 1.00 m/s, 1.25 m/s, and 1.50 m/s) and the inter-speed mode

To verify the net PF moment prediction performance of the proposed sEMG-US imaging-driven HNM and compare the new HNM to both sEMG- and US imaging-driven HNMs, the three calibrated HNM categories, shown in Fig. 8, were applied to walking scenarios of five different speeds. Similarly, $BM-RMSE$, $N-RMSE$, and $R^{2}$ values between the predicted and ID-calculated net PF moments were evaluated and compared. Due to the space limitation, the detailed *RMSE* and $R^{2}$ values at each walking speed on individual participant in prediction can be found in Additional file 1: Tables S2 to Table S4. The summarized results in Fig. 9 show the mean and one SD of $BM-RMSE$, $N-RMSE$, and $R^{2}$ values in prediction at each of five speeds by applying three HNM categories. The subplots from the first column to the fifth column represent the applied calibrated HNMs were from five single-speed modes, while the subplots from the sixth column represent the inter-speed mode. In each subplot, the $x-$axis labels represent the prediction scenarios at five different speeds. Table 3 summaries the mean values of $BM-RMSE$, $N-RMSE$, and $R^{2}$ in the prediction by using the proposed sEMG-US imaging-driven HNM, where the bold numbers represent the cases that the calibration speed mode is the same as the prediction speed scenario, which always results in the lowest *RMSE* and highest $R^{2}$ values. From Fig. 9 and Table 3, we observed that $BM-RMSE$, $N-RMSE$, and $R^{2}$ values are optimal when the applied calibrated single-speed mode is close to the current prediction speed scenario, regardless of HNM categories. Furthermore, the calibrated inter-speed mode can effectively constrain the $BM-RMSE$, $N-RMSE$, and $R^{2}$ values within a small variation range among all prediction speed scenarios, regardless of HNM categories.

The results of two-way repeated-measure ANOVA show that in prediction $BM-RMSE$ values are significantly affected by the calibration speed mode (main effect, $p<0.001$, $\eta _{p}^{2}=0.104$) and applied HNM category (main effect, $p<0.001$, $\eta _{p}^{2}=0.141$), $N-RMSE$ values are significantly affected by the calibration speed mode (main effect, $p<0.001$, $\eta _{p}^{2}=0.101$) and applied HNM category (main effect, $p<0.001$, $\eta _{p}^{2}=0.164$), and also $R^{2}$ values are significantly affected by the calibration speed mode (main effect, $p<0.001$, $\eta _{p}^{2}=0.103$) and applied HNM category (main effect, $p<0.001$, $\eta _{p}^{2}=0.164$). The pairwise comparisons show that across all prediction scenarios with every speed mode calibration, the proposed sEMG-US imaging-derived HNM could significantly reduce the $BM-RMSE$ values by 14.49% ($p=0.029$, $d=-0.348$) and 36.94% ($p<0.001$, $d=-0.930$), significantly reduce the $N-RMSE$ values by 14.58% ($p=0.012$, $d=-0.389$) and 36.79% ($p<0.001$, $d=-1.021$), and increase the $R^{2}$ values by 9.71% ($p=0.023$, $d=0.713$) and 17.86% ($p<0.001$, $d=1.010$) compared to sEMG-driven and US imaging-driven HNMs, respectively. Furthermore, the pairwise comparisons also show that across all prediction scenarios with every HNM category, the inter-speed mode calibration could reduce the $BM-RMSE$ values by 38.74% ($p<0.001$, $d=-0.922$), 18.86% ($p=0.180$, $d=-0.590$), 8.87% ($p=0.826$, $d=-0.237$), 9.84% ($p=0.785$, $d=-0.299$), and 26.57% ($p=0.004$, $d=-0.836$), reduce the $N-RMSE$ values by 35.73% ($p<0.001$, $d=-1.002$), 17.20% ($p=0.201$, $d=-0.403$), 9.11% ($p=0.881$, $d=-0.254$), 11.44% ($p=0.715$, $d=-0.326$), and 28.08% ($p<0.001$, $d=-0.817$), and increase the $R^{2}$ values by 16.76% ($p<0.001$, $d=0.810$), 5.33% ($p=0.307$, $d=0.342$), 0.40% ($p=0.896$, $d=0.044$), 0.31% ($p=0.921$, $d=0.033$), and 6.51% ($p=0.127$, $d=0.487$) compared to single-speed mode calibration at 0.50 m/s, 0.75 m/s, 1.00 m/s, 1.25 m/s, and 1.50 m/s, respectively.

**Table 3:** Metrics of $BM-RMSE$, $N-RMSE$, and $R^{2}$ mean values of the net PF moment prediction across all participants by using the proposed sEMG-US imaging-driven HNM

$BM-RMSE$		Prediction scenario (m/s)				
		0.50	0.75	1.00	1.25	1.50
Calibration	0.50 m/s	**0.119**	0.154	0.221	0.276	0.333
	0.75 m/s	0.151	**0.121**	0.171	0.221	0.284
	1.00 m/s	0.216	0.162	**0.127**	0.176	0.275
	1.25 m/s	0.254	0.182	0.159	**0.148**	0.214
	1.50 m/s	0.271	0.226	0.215	0.214	**0.155**
	Inter-speed	0.161	0.126	0.150	0.163	0.217
$$N-RMSE$$						
Calibration	0.50 m/s	**8.36**	9.78	12.72	14.66	16.35
	0.75 m/s	10.75	**7.75**	9.75	11.71	14.04
	1.00 m/s	15.29	10.29	**7.39**	9.39	13.54
	1.25 m/s	17.97	11.52	9.01	**7.89**	10.87
	1.50 m/s	19.22	14.33	12.36	11.44	**7.71**
	Inter-speed	11.40	8.01	8.60	8.65	10.84
$$R^{2}$$						
Calibration	0.50 m/s	**0.909**	0.895	0.876	0.854	0.794
	0.75 m/s	0.863	**0.943**	0.930	0.908	0.845
	1.00 m/s	0.838	0.933	**0.948**	0.920	0.855
	1.25 m/s	0.809	0.912	0.928	**0.940**	0.904
	1.50 m/s	0.769	0.855	0.877	0.899	**0.946**
	Inter-speed	0.881	0.933	0.943	0.935	0.899

The diagonal elements with bold numbers represent the situations that the calibration speed mode is the same as the prediction speed scenario. ($BM-RMSE$ unit: N$\cdot$m/kg, $N-RMSE$ unit: %)

## Discussions

For the first time, this study investigated net ankle joint PF moment continuous prediction during the walking stance phase based on an HNM approach that combines both sEMG-induced and US imaging-induced muscle activation components. We defined this approach as sEMG-US imaging-driven HNM for the net PF moment prediction. The modified HNM was validated with quasi-periodic data, including sEMG signals and US imaging-derived MT from plantarflexors, ankle joint kinematics, and kinetics measurements from multiple treadmill walking stance phases gait cycles at five different speeds. We focused on two plantarflexor muscles, e.g., LGS and SOL, when characterizing the PF function during walking. The basic objective was to establish a modified HNM for either LGS or SOL muscles, including (1) a weighted muscle activation model component, (2) a muscle-tendon unit geometry model component, and (3) a muscle dynamic contraction model component. This study also investigated the effects of HNM categories (sEMG-, US imaging-, and sEMG-US imaging-driven) and calibration speed modes (single-speed modes and inter-speed mode) on the net PF moment prediction performance during the walking stance phase. The ID-calculated net PF moment worked as the benchmark in both calibration and prediction. The results support our hypothesis that compared to sEMG-driven and US imaging-driven HNMs, the sEMG-US imaging-driven HNM accurately predicts the net PF moment. Further, the HNMs, when calibrated with the inter-speed mode, robustly predict the net PF moment.

The evaluation of the proposed HNMs’ calibration and prediction performance was based on three criteria: $BM-RMSE$, $N-RMSE$, and $R^{2}$. In general, as reported in [58], the results were considered excellent if the $N-RMSE$ values were smaller than 15%. For the calibration, the results in Fig. 7 presented a good calibration performance by using each of three HNMs on Sub05. Furthermore, from results across participants shown in Fig. 8, we observed that the mean $N-RMSE$ values were all less than 10.92% regardless of the calibration speed modes and applied HNM categories. Primarily they were less than 8.30% irrespective of the calibration speed modes when applying the proposed sEMG-US imaging-driven HNM. For the prediction, as shown in Fig. 9 and Table 3, by applying the inter-speed mode calibrated sEMG-US imaging-driven HNM, the $N-RMSE$ mean values were all less than 11.40% throughout those five walking speeds scenarios, which validated the prediction results were all excellent. Therefore, both calibration and prediction results present consistent findings that the $N-RMSE$ values can be constrained within a satisfactorily small region by using the proposed sEMG-US imaging-driven HNM with the inter-speed mode calibration.

The superior net PF torque prediction of the sEMG-US imaging-driven HNM can be mainly due to the reason that the fused signals capture complementary mechanical and neural aspects of the muscle contraction [25]. information [25]. Specifically, sEMG signals measure electric potentials generated by muscle motor units. The amplitude and density of sEMG signals linearly correlate with the number of firing neurons, which offers a physical measurement of the microphysiological response [59]. US imaging directly visualizes muscle change at the macrophysiological performance [60], when the same group of neural motor units is activated. Thus, sEMG features and US imaging features provide information from electrical and mechanical aspects, respectively, in response to the same physiological stimulus.

As a preliminary study of the sEMG-US imaging-driven HNM for walking on a treadmill across multiple speeds, the results are promising and can help overcome the challenges of joint motion intent detection in volitional control of assistive devices. However, there are still some limitations in the current study. First of all, only a few US imaging-derived parameters, MT from both LGS and SOL, measured muscle activation levels indirectly (a macrophysiological perspective). Other US-derived parameters, like FL and PA [21, 25, 27, 35], of the MTU geometry model may further enrich the neuromuscular model. A potential difficulty here is the inability to capture a larger region of interest that contains entire muscle fascicles, mainly due to the small dimension of our US transducer. Another difficulty is to continuously track the muscle fascicles of the plantarflexors within the visualized region of interest during dynamic tasks, like walking. If always visualized, more geometry parameters from US imaging signals will likely further improve the HNM accuracy. Second, the US imaging-derived muscle activation was defined as a normalization function in (4). We also assumed a linear monotonic relationship between the MT of LGS or SOL and net PF moment change during the stance phase in Table 1. However, in [29, 61], it was observed that despite an increase in MT of the LGS and SOL muscles during the stance phase, their muscle activations decreased in the last 10 to 20% of the stance phase. Also, in fast walking, MT of SOL was observed to decrease in the first 10% of stance, while the muscle activation increased. The above inconsistency may result from the difference in experimental setup, sEMG sensor or US transducer placement on the targeted muscles, MT tracking approach from US imaging signals, etc. In future work, a further comparison will be conducted to evaluate the muscle geometry parameters change and sEMG signals change during the same walking tasks. As detailed in the pre-processing data section, a key-frame correction [55] was applied to reduce the gait cycle-related drift of MT tracking results significantly. However, a subjective error in the manual operation is inevitable. Therefore, it was not practical to altogether remove the gait cycle-related drift of US imaging-derived MT. This work has extended the preliminary results of voluntary isometric ankle PF studies [34, 35] to dynamic walking studies on non-disabled participants. Further experiments on patients with impaired plantarflexors are needed to verify the practicability of the proposed sEMG-US imaging-driven HNM for net ankle joint PF moment prediction. We also observed that the calibrated HNMs with the inter-speed mode increased the net PF moment prediction robustness across different speed scenarios. The development of inter-speed HNM modeling is crucial because it will potentially allow a consistently accurate prediction performance of the net PF moment across multiple walking speeds. Furthermore, it will enable predicting and analyzing the dynamics of a more extensive set of walking speeds than was possible with a single-speed mode, resulting in a deeper understanding of the neuromuscular dynamics during the human walking stance phase. Finally, it may open up to the development of robust neuromuscular human-machine interfaces for the simultaneous and proportional control of wearable assistive devices such as powered orthoses and prostheses under different walking speeds.

In this preliminary study, although the combination of US imaging and sEMG data in the model improved the prediction of the ankle moment, it was unknown if the prediction was better only because in this combination the solution space was higher by adding parameters in the optimization problem. To address this ambiguity, we have conducted additional model calibration and prediction procedures for comparison, where the time sequence of the US imaging-derived muscle activation was replaced by a randomly generated signal between 0 and 1. In this way, we could reproduce the same solution space just like the proposed sEMG-US imaging-driven HNM, so we would be able to investigate if the prediction benefits still exist or not. We took a representative participant as an example, and present the calibration performance and prediction performance by using five different HNMs, as shown in the Additional file 1: Fig. S18. It was observed that when the random signal (RS)-derived muscle activation levels of LGS and SOL muscles were introduced, although the solution space was increased, just like the proposed approach to fuse sEMG and US imaging signals, the calibration and prediction performance were both deteriorated when compared to the case where only sEMG-derived muscle activation levels were used. Therefore, based on the additional analyses, we believe that the better net plantarflexion moment prediction by using the proposed sEMG-US imaging-driven HNM was not only because in this combination the solution space was higher by adding parameters in the optimization problem, but more importantly, the US imaging-derived muscle thickness feature introduced complementary mechanical deformation characteristics during the muscle contraction, which dominantly resulted in the better prediction performance.

### Limitation and future work

The key point to implement the proposed sEMG-US imaging-driven HNM is to find the allocation gain $\delta _{i}$ for both LGS and SOL muscles through solving the nonlinear least square optimization problem. However, one possible limitation of this optimization process is that the allocation gains are speed-dependent and subject-dependent, which means that the optimal values of those two allocation gains vary along with the model calibrations when using data collected from different walking speeds on the same participant or from different participants at the same walking speed. So it is relatively challenging to generate a constant allocation gain for either LGS or SOL muscles across multiple speeds or general participants. The US imaging-derived muscle thickness signal is potentially sensitive to the position and orientation of the US transducer relative to the shank segment, which may affect the prediction accuracy of the proposed approach. In the current study, we did not track the position and orientation of the US transducer relative to the shank segment due to the assumption that the relative motion of the US transducer is negligible because a customized US transducer holder with an arc structure and elastic velcro straps were used to stabilize the position and orientation of the US transducer during the dynamic walking tasks. However, one possible limitation of this study is that the assumption may not hold for a faster walking speed. Therefore, a further study would be to investigate the effect of the relative position and orientation change between the US transducer and the shank segment to the prediction accuracy of the proposed sEMG-US imaging-driven HNM approach.

Another limitation of the current study is that we only considered the agonist LGS and SOL muscles to predict the ankle joint net PF moment, and the effects of the MGS and TA muscles were not considered. Although in the experiments we collected the sEMG signals from LGS, MGS, SOL, and TA muscles, ultrasound imaging data was collected from the LGS and SOL muscles only. Therefore, the fusion results were only based on these two muscles. The main reasons that we did not consider the effects from the MGS and TA muscles are (1) existing evidence suggests that sEMG signals from LGS and MGS are comparable [62], and similar results in the current study also can be seen in the Additional file 1: Fig. S19; (2) during the walking stance phase, plantarflexor muscles (LGS and SOL) are the dominant muscles that generate the net ankle joint moment. Results of the processed sEMG signals showed that the activation of the TA muscle are relatively small during the walking stance phase compared to the plantarflexor muscles, as can be observed in the Additional file 1: Fig. S19. However, even though the TA muscle activation is low during the walking stance phase, the effect from the passive contraction from this antagonist muscle needs to be further investigated in future work.

## Conclusion

This paper investigated the feasibility of an sEMG-US imaging-driven HNM to predict net ankle joint PF moment during the stance phase across multiple walking speeds. The results showed that on average, the net PF moment prediction *RMSE*, normalized to peak net PF moment/body mass, was significantly reduced when using the sEMG-US imaging-driven HNM, compared to sEMG-driven and US imaging-driven HNMs. The results also showed that the calibrated HNMs with the inter-speed mode were more robust for net PF moment prediction at different speed scenarios than HNMs with single-speed modes. The improved net ankle joint PF moment prediction during the dynamic walking tasks at different speeds could potentially lead to improved volitional control of assistive devices with more advanced and intelligent algorithms.

## Supplementary Information

**Additional file 1: Table S1.** Participants’ anthropometric characteristics and peak net PF moment during treadmill walking at each speed. **Table S2.** HNMs prediction performance under single-speed modes and inter-speed mode on Sub01. (RMSE unit: Nm). **Table S3.** HNMs prediction performance under single-speed modes and inter-speed mode on Sub02. (RMSE unit: Nm). **Table S4.** HNMs prediction performance under single-speed modes and inter-speed mode on Sub03. (RMSE unit: Nm). **Table S5.** HNMs prediction performance under single-speed modes and inter-speed mode on Sub04. (RMSE unit: Nm). **Table S6.** HNMs prediction performance under single-speed modes and inter-speed mode on Sub05. (RMSE unit: Nm). **Figure S1.** Right ankle joint kinematics and kinetics results from Participant Sub01. **Figure S2.** Right ankle joint kinematics and kinetics results from Participant Sub02. **Figure S3.** Right ankle joint kinematics and kinetics results from Participant Sub03. **Figure S4.** Right ankle joint kinematics and kinetics results from Participant Sub04. **Figure S5.** Right ankle joint kinematics and kinetics results from Participant Sub05. **Figure S6.** sEMG raw data and processed data from both LGS and SOL muscles during 20 seconds walking experiments on Participant Sub01. **Figure S7.** sEMG raw data and processed data from both LGS and SOL muscles during 20 seconds walking experiments on Participant Sub02. **Figure S8.** sEMG raw data and processed data from both LGS and SOL muscles during 20 seconds walking experiments on Participant Sub03. **Figure S9.** sEMG raw data and processed data from both LGS and SOL muscles during 20 seconds walking experiments on Participant Sub04. **Figure S10.** sEMG raw data and processed data from both LGS and SOL muscles during 20 seconds walking experiments on Participant Sub05. **Figure S11.** US imaging-derived MT and processed sEMG signals during the walking stance phase from Sub01. **Figure S12.** US imaging-derived MT and processed sEMG signals during the walking stance phase from Sub02. **Figure S13.** US imaging-derived MT and processed sEMG signals during the walking stance phase from Sub03. **Figure S14.** US imaging-derived MT and processed sEMG signals during the walking stance phase from Sub04. **Figure S15.** US imaging-derived MT and processed sEMG signals during the walking stance phase from Sub05. **Figure S16.** The Ultratrack toolbox interface with US imaging-derived MT tracking procedures and results without key-frame correction on Participant Sub07 walking at 1.00 m/s. **Figure S17.**The Ultratrack toolbox interface with US imaging-derived MT tracking procedures and results with key-frame correction on Participant Sub07 walking at 1.00 m/s. **Figure S18.** Exampled calibration and prediction performance on Participant Sub01 by using five HNM categories. The blue, red, orange, purple, and green bars represent the results by using the sEMG-, US imaging-, sEMG-US imaging-, random signal (RS)-, and sEMG-RS-driven HNMs under both single-speed modes and inter-speed mode. (a) Calibration RMSE and prediction RMSE values under different speed modes. (b) Calibration R-square and prediction R-square values under different speed modes. **Figure S19.** Exampled results of the ankle joint net moment and processed sEMG signals from LGS, MGS, SOL, and TA muscles throughout the recorded walking duration. Data are collected during the treadmill walking experiments on Sub01, where muscle activation levels from LGS and MGS muscles are comparable, and the activation level of the TA muscle is relatively small during the walking stance phase. (a) Walking speed at 0.5 m/s. (b) Walking speed at 0.75 m/s.

**Additional file 2.** The LGS and SOL muscles' thickness tracking results from sequential US images on Participant Sub01 during treadmill walking at 0.50 m/s.

**Additional file 3.** The LGS and SOL muscles' thickness tracking results from sequential US images on Participant Sub01 during treadmill walking at 0.75 m/s.

**Additional file 4.** The LGS and SOL muscles' thickness tracking results from sequential US images on Participant Sub01 during treadmill walking at 1.00 m/s.

**Additional file 5.** The LGS and SOL muscles' thickness tracking results from sequential US images on Participant Sub01 during treadmill walking at 1.25 m/s.

**Additional file 6.** The LGS and SOL muscles' thickness tracking results from sequential US images on Participant Sub01 during treadmill walking at 1.50 m/s.

## Data Availability

The datasets used and/or analyzed during the current study are available from the corresponding author on reasonable request.

## Abbreviations

GRF: Ground reaction force
ID: inverse dynamics
HNM: Hill-type neuromuscular model
EMG: Electromyography
sEMG: Surface electromyography
PA: Pennation angle
FL: Fascicle length
MT: Muscle thickness
MGS: Medial gastrocnemius
LGS: Lateral gastrocnemius
SOL: Soleus
PF: Plantarflexion
EMD: Electromechanical delay
MTU: Muscle-tendon unit
IRB: Institutional Review Board
B-mode: Brightness mode
ANOVA: Analysis of variance
HSD: Honestly significant difference test
RMSE: Root mean square error
N-RMSE: Root mean square error normalized to peak value
BM-RMSE: Root mean square error normalized to body mass
$${\eta _p}^2$$: Effect size
SD: Standard deviation
*R*: Correlation coefficient
$R^2$: Coefficient of determination
*d*: Cohen’s *d*
US: Ultrasound
