# Supplementary material for: Fused ultrasound and electromyography-driven neuromuscular model to improve plantarflexion moment prediction across walking speeds
Source: J Neuroeng Rehabil. 2022 Aug 9;19:86. doi: 10.1186/s12984-022-01061-z (PMC9361708; doi:10.1186/s12984-022-01061-z)
Supplement: Supplementary file 1 — Additional file 1: Table S1. Participants’ anthropometric characteristics and peak net PF moment during treadmill walking at each speed. Table S2. HNMs prediction performance under single-speed modes and inter-speed mode on Sub01. (RMSE unit: Nm). Table S3. HNMs prediction performance under single-speed modes and inter-speed mode on Sub02. (RMSE unit: Nm). Table S4. HNMs prediction performance under single-speed modes and inter-speed mode on Sub03. (RMSE unit: Nm). Table S5. HNMs prediction performance under single-speed modes and inter-speed mode on Sub04. (RMSE unit: Nm). Table S6. HNMs prediction performance under single-speed modes and inter-speed mode on Sub05. (RMSE unit: Nm). Figure S1. Right ankle joint kinematics and kinetics results from Participant Sub01. Figure S2. Right ankle joint kinematics and kinetics results from Participant Sub02. Figure S3. Right ankle joint kinematics and kinetics results from Participant Sub03. Figure S4. Right ankle joint kinematics and kinetics results from Participant Sub04. Figure S5. Right ankle joint kinematics and kinetics results from Participant Sub05. Figure S6. sEMG raw data and processed data from both LGS and SOL muscles during 20 seconds walking experiments on Participant Sub01. Figure S7. sEMG raw data and processed data from both LGS and SOL muscles during 20 seconds walking experiments on Participant Sub02. Figure S8. sEMG raw data and processed data from both LGS and SOL muscles during 20 seconds walking experiments on Participant Sub03. Figure S9. sEMG raw data and processed data from both LGS and SOL muscles during 20 seconds walking experiments on Participant Sub04. Figure S10. sEMG raw data and processed data from both LGS and SOL muscles during 20 seconds walking experiments on Participant Sub05. Figure S11. US imaging-derived MT and processed sEMG signals during the walking stance phase from Sub01. Figure S12. US imaging-derived MT and processed sEMG signals during the walking stance phase from Sub02. Fi [file 12984_2022_1061_MOESM1_ESM.pdf]

SUPPLEMENTAL MATERIALS

In the supplemental materials, more details are included to support the results presented in the paper. The reminders are given here. The full results of the right ankle joint's angular position, velocity, and net plantarflexion moment from inverse kinematics and inverse dynamics, the raw sEMG signals from both lateral gastrocnemius (LGS) and soleus (SOL) muscles, and the neuromuscular features from both LGS and SOL muscles across all walking stance cycles at each walking speed (out of five from 0.50 m/s to 1.50 m/s) on representative participants are shown in section A. The muscle thickness (MT) tracking results from ultrasound imaging on representative participants are shown in section B. The prediction root mean square error (RMSE) and R^2 values under the single-speed modes and inter-speed mode using the sEMG-US imaging-, sEMG-, and US imaging-driven HNMs on each participant are used summarized in section C. Additional results for supporting discussion section are included in section D. Examples of the LGS and SOL muscles' thickness tracking results from sequential US images on Participant Sub01 were recorded as videos in separate supplemental materials. A commercial toolbox UltraTrack was applied to conduct the muscle thickness tracking, and key-frame correction was applied to minimize the time drift along with multiple gait cycles.

A. Ankle joint kinematics and kinetics results, LGS and SOL muscles' raw sEMG signals, and sEMG- and US imaging-derived neuromuscular features

In this section, first, the participants' anthropometric characteristics and peak net PF moment during the walking stance phase at each speed are summarized in Table S-I. Then, the data from five representative participants (Sub01 – Sub05) are presented to save space. First, the time sequences of ankle joint kinematics and kinetics data were distributed along the normalized gait cycle from 0 % (heel-strike) to 100 % (subsequent heel-strike), and the results from representative participants are shown in Fig. S1 to Fig. S5.

Table S-I Participants' anthropometric characteristics and peak net PF moment during treadmill walking at each speed.

Participant	Height (m)	Body mass (kg)	Age (years old)	Peak net PF moment (Nm)				
				0.50 m/s	0.75 m/s	1.00 m/s	1.25 m/s	1.50 m/s
Sub01	1.93	108.0	24	155.50	174.57	187.97	206.32	217.27
Sub02	1.64	54.0	24	75.13	89.32	96.94	103.25	112.62
Sub03	1.76	84.0	30	124.31	129.44	147.59	147.19	149.27
Sub04	1.77	82.0	27	114.42	132.14	138.30	152.14	165.35
Sub05	1.77	62.0	22	90.04	97.25	100.71	109.68	123.18
Sub06	1.73	70.0	26	73.43	81.37	100.78	107.12	110.08
Sub07	1.62	56.0	21	105.59	125.36	132.33	145.88	170.69
Sub08	1.77	71.0	29	92.13	100.36	114.17	128.77	136.52
Sub09	1.70	75.0	25	103.81	116.22	127.34	137.54	148.12
Sub10	1.75	68.0	28	98.32	110.14	121.14	130.75	140.93
Mean	1.74	73.0	25.6	103.26	115.62	126.73	136.86	147.40
SD	0.08	15.8	2.9	24.28	26.87	27.44	30.01	31.80

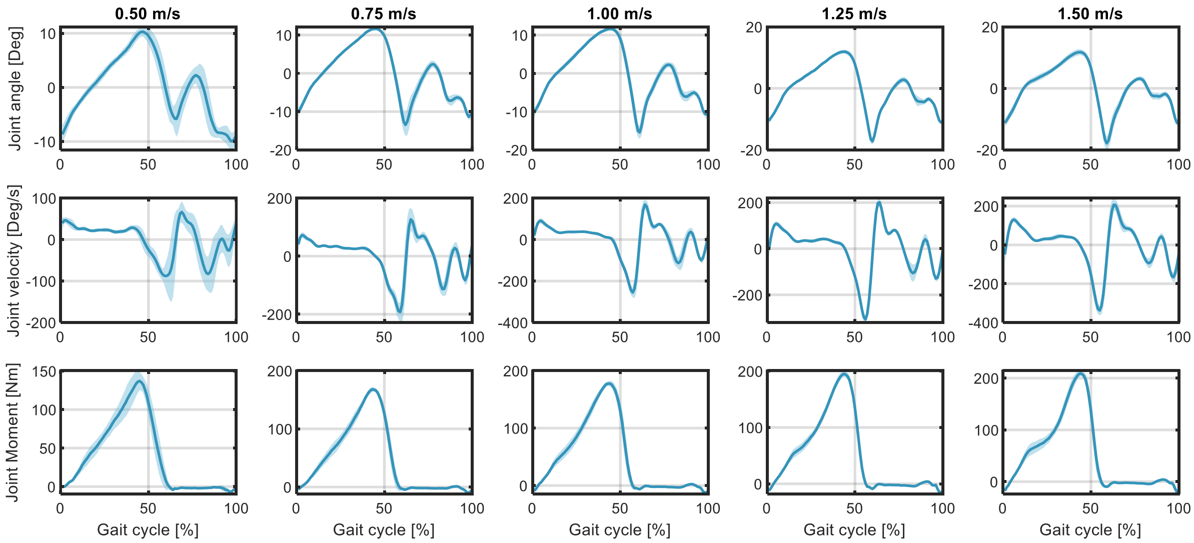

Figure S1 Right ankle joint kinematics and kinetics results from Participant Sub01

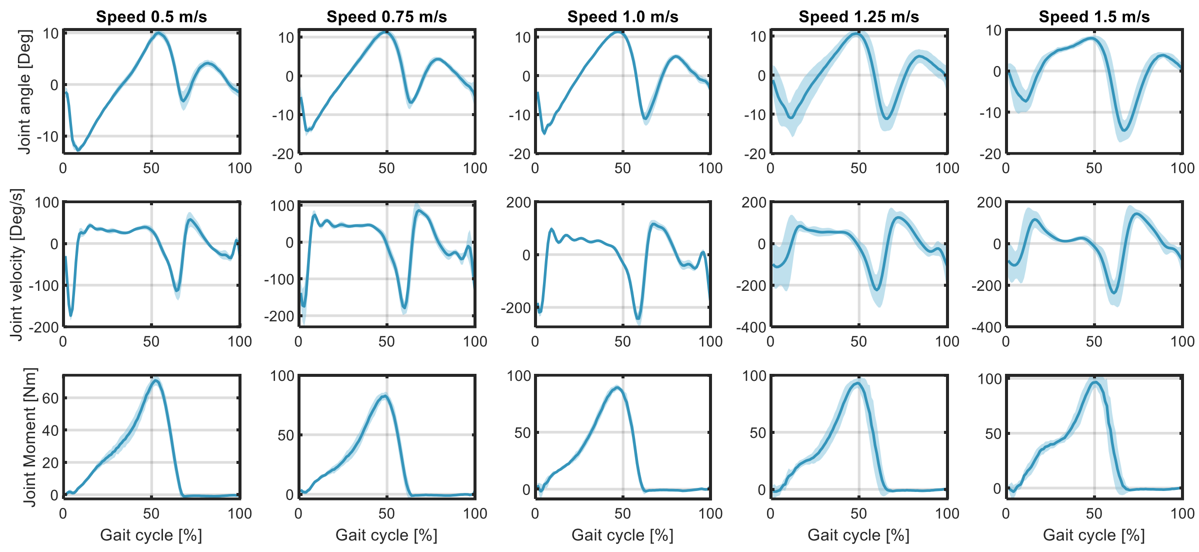

Figure S2 Right ankle joint kinematics and kinetics results from Participant Sub02

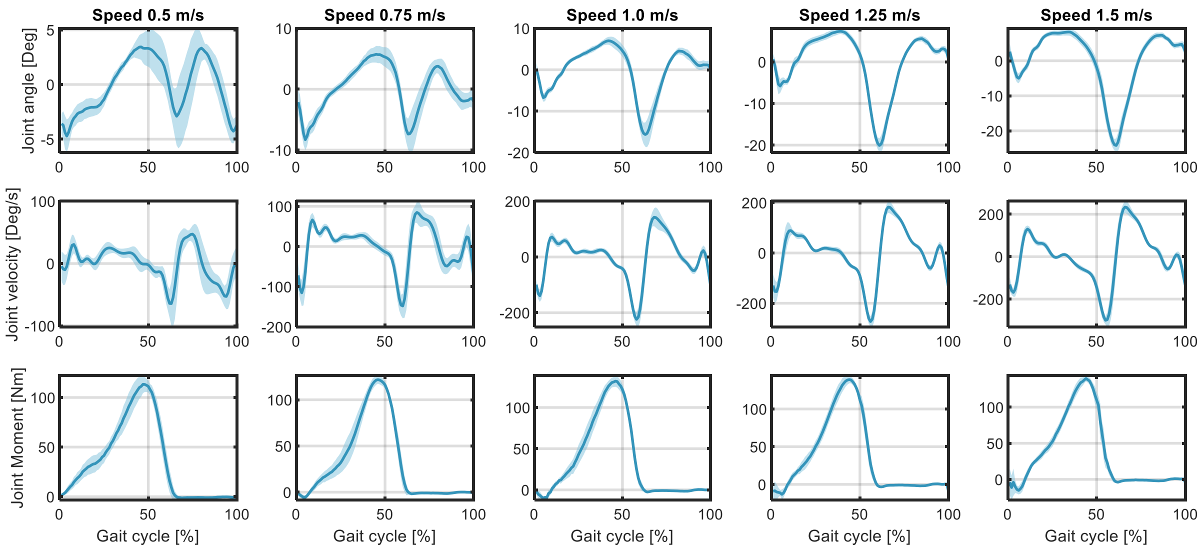

Figure S3 Right ankle joint kinematics and kinetics results from Participant Sub03

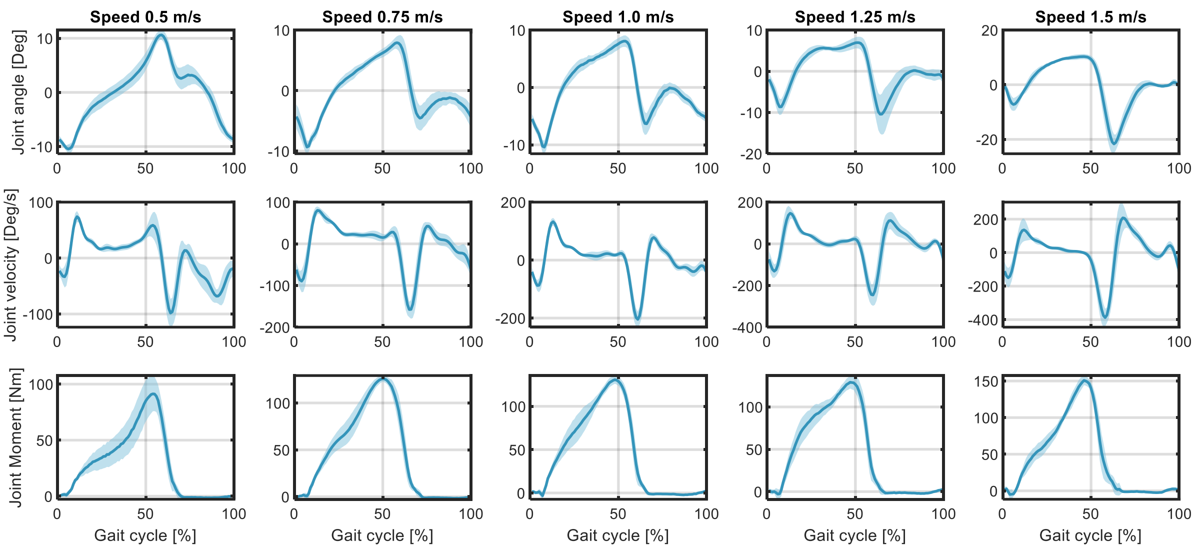

Figure S4 Right ankle joint kinematics and kinetics results from Participant Sub04

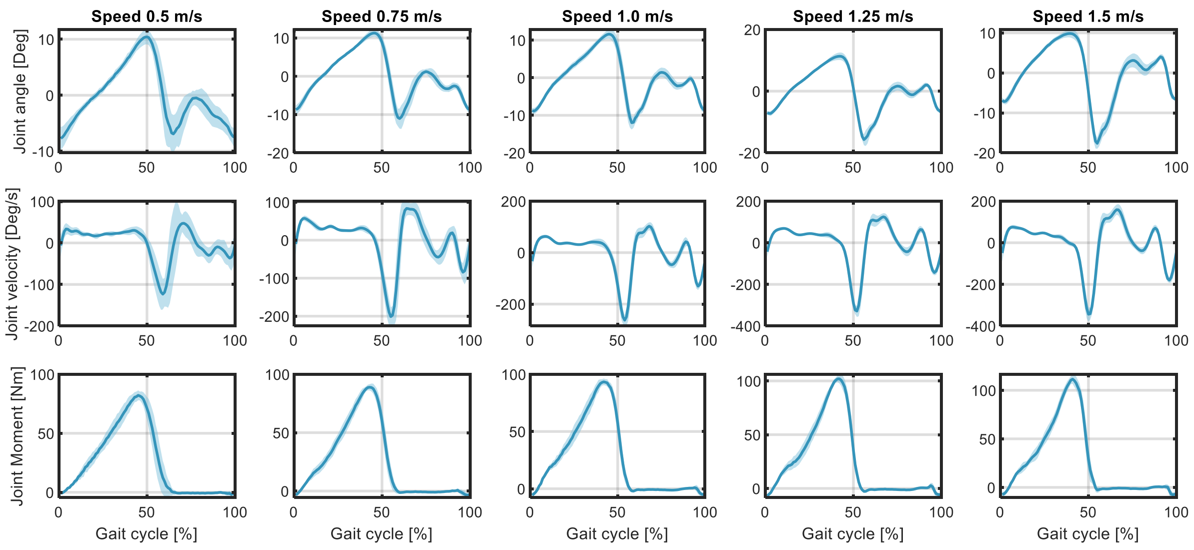

Figure S5 Right ankle joint kinematics and kinetics results from Participant Sub05

The corresponding sEMG raw data and processed data of both LGS and SOL muscles on representative participants are presented in Fig. S6 to Fig. S10.

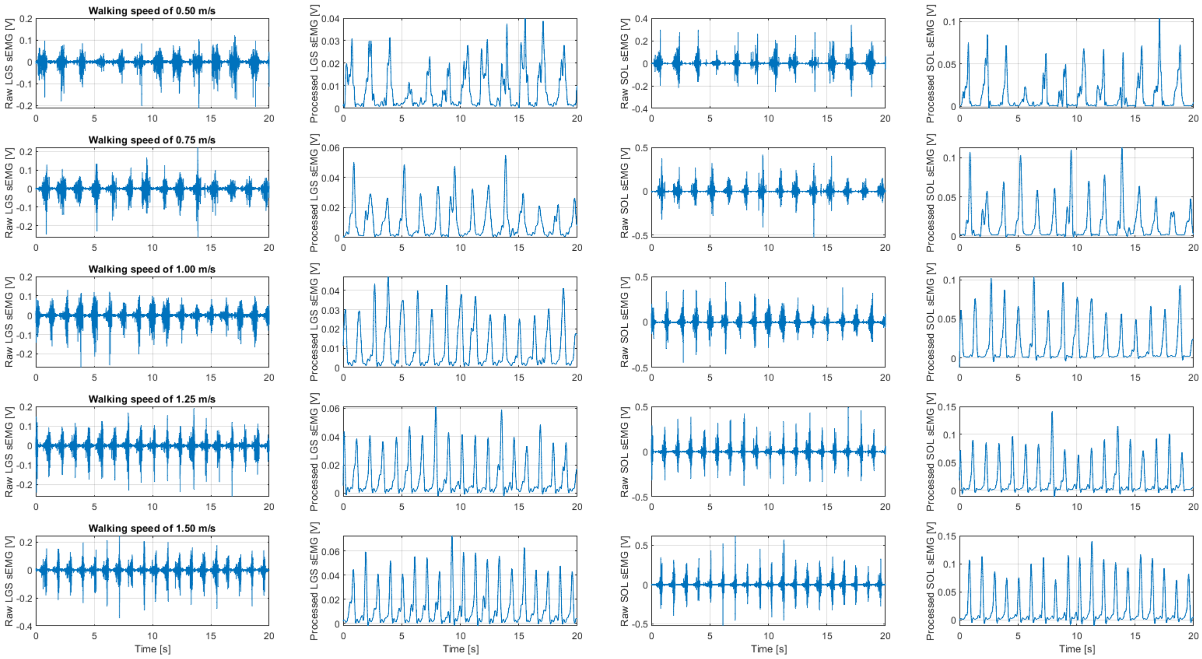

Figure S6 sEMG raw data and processed data from both LGS and SOL muscles during 20 seconds walking experiments on Participant Sub01

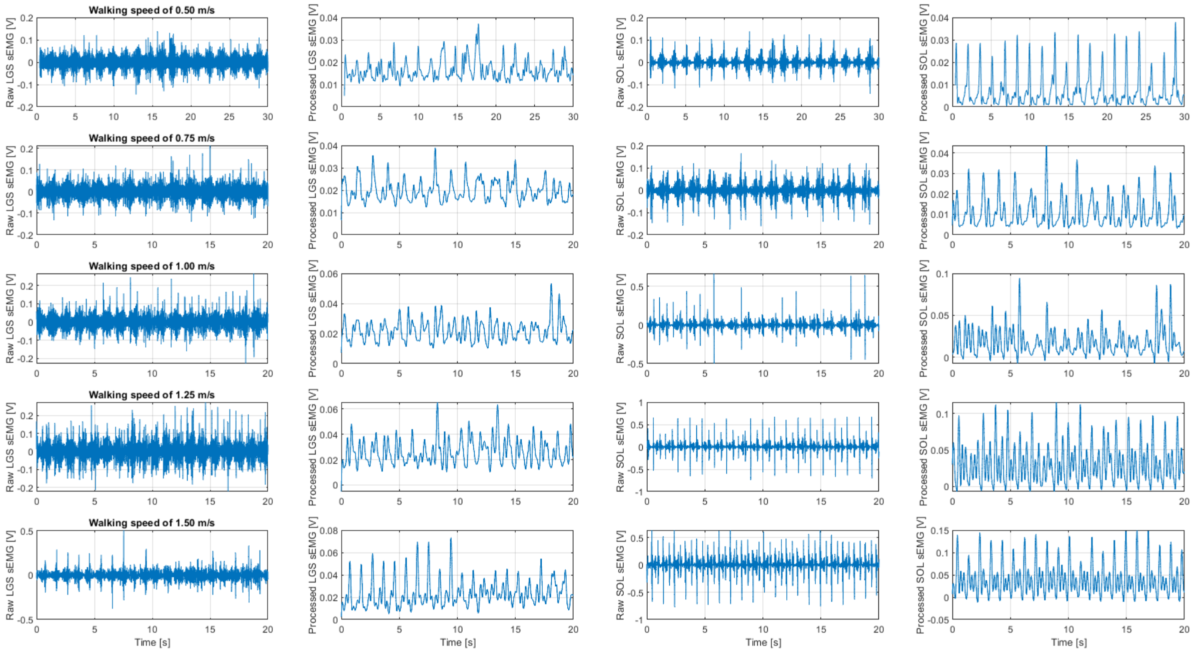

Figure S7 sEMG raw data and processed data from both LGS and SOL muscles during 20 seconds walking experiments on Participant Sub02

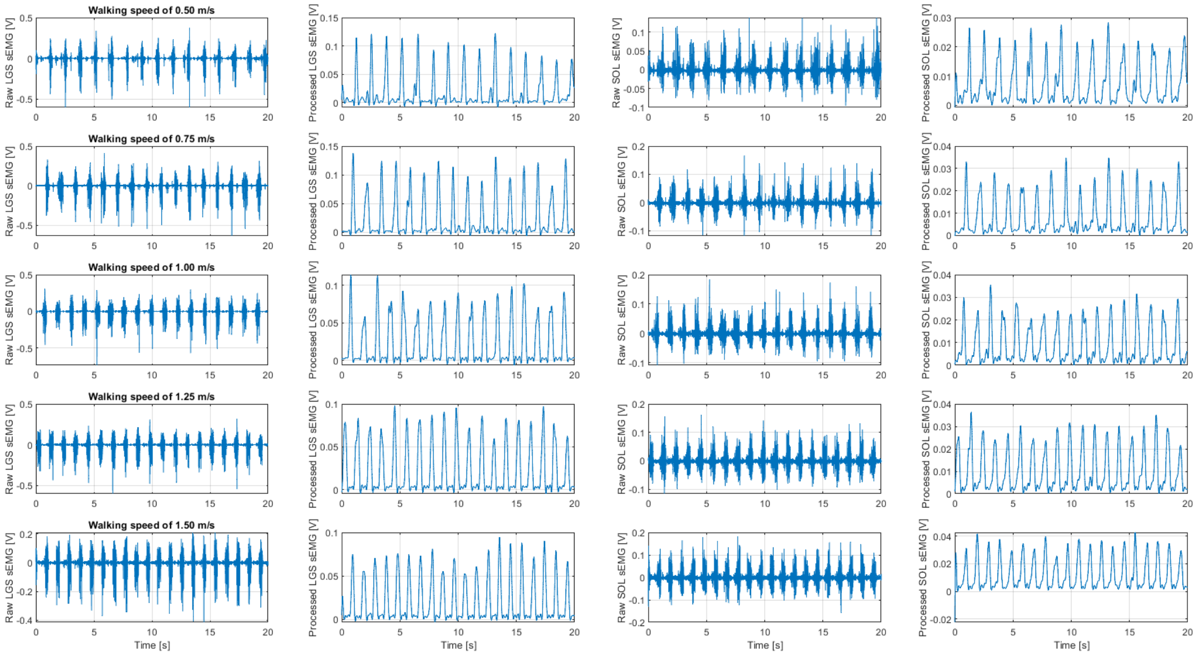

Figure S8 sEMG raw data and processed data from both LGS and SOL muscles during 20 seconds walking experiments on Participant Sub03

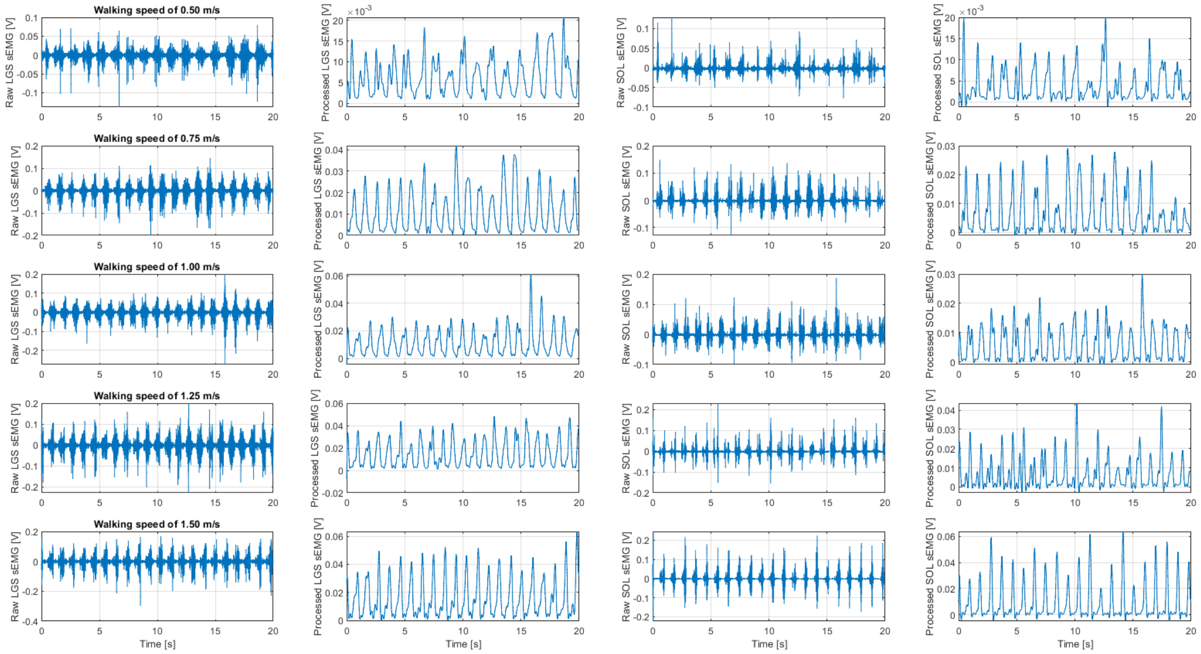

Figure S9 sEMG raw data and processed data from both LGS and SOL muscles during 20 seconds walking experiments on Participant Sub04

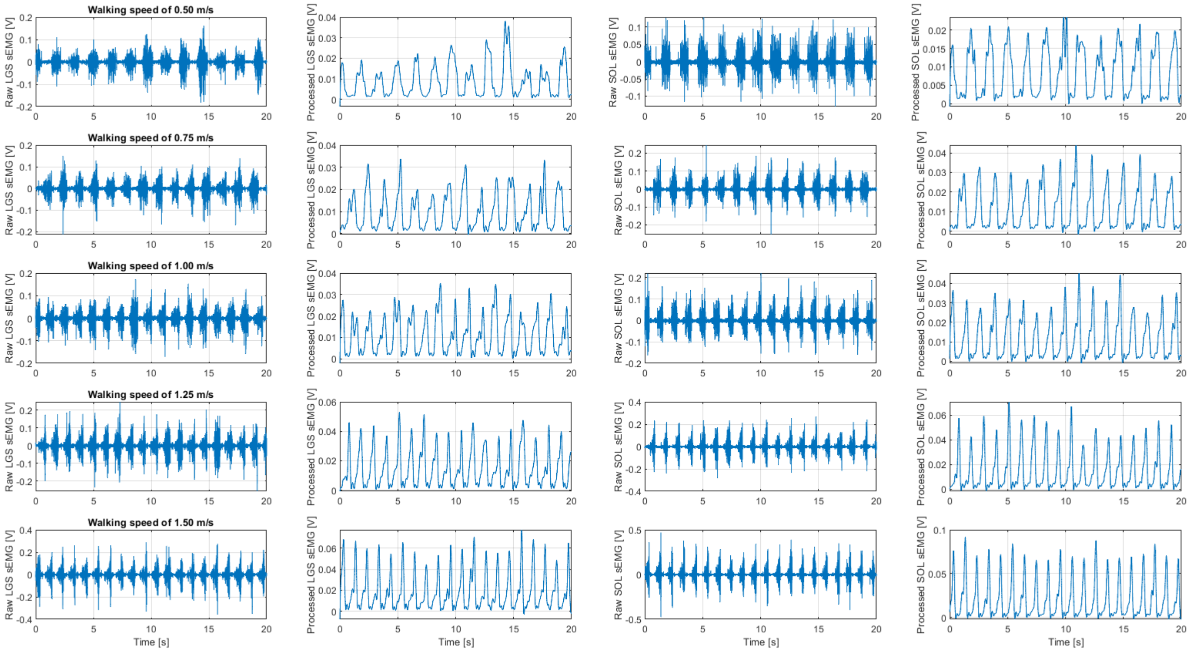

Figure S10 sEMG raw data and processed data from both LGS and SOL muscles during 20 seconds walking experiments on Participant Sub05

The corresponding LGS and SOL muscle thickness changes extracted from US images and processed sEMG changes were distributed along the normalized stance cycle from 0 % (heel-strike) to 100 % (toe-off), and the results from those representative participants are shown in Fig. S11 to Fig. S15.

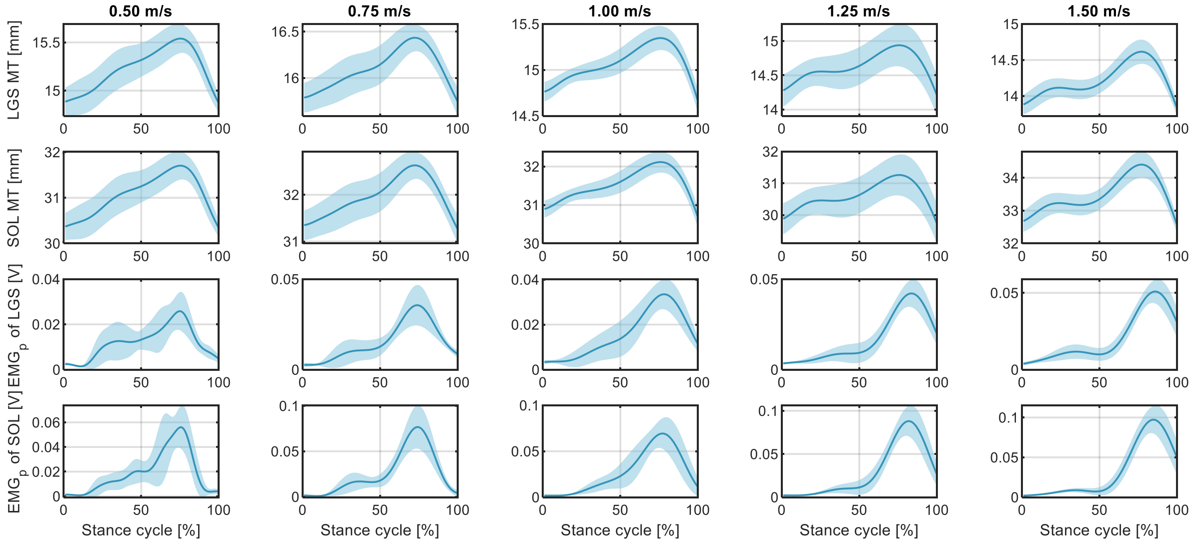

Figure S11 US imaging-derived MT and processed sEMG signals during the walking stance phase from Sub01

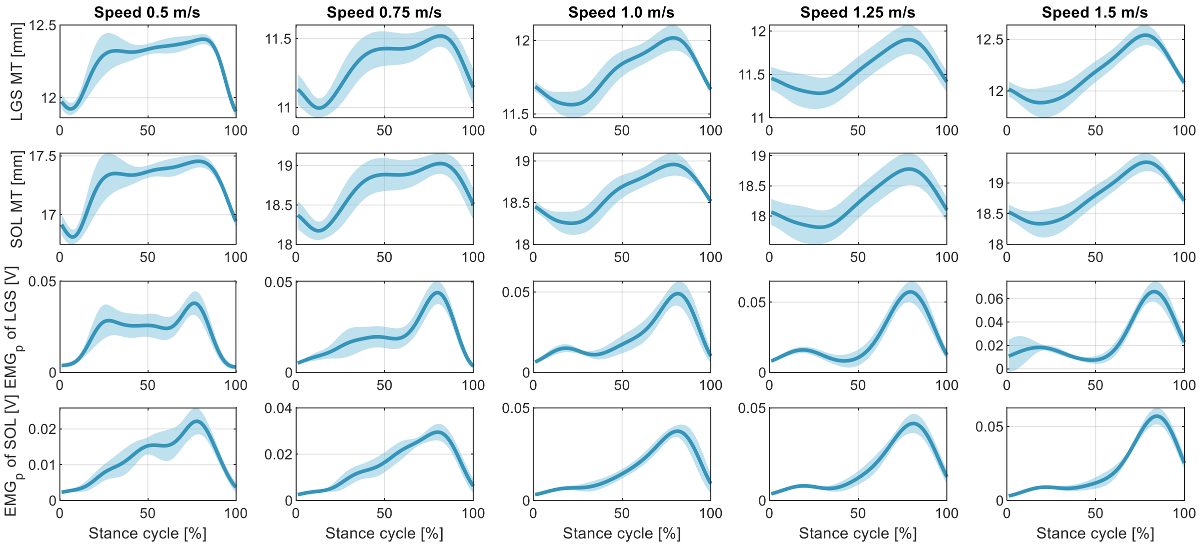

Figure S12 US imaging-derived MT and processed sEMG signals during the walking stance phase from Sub02

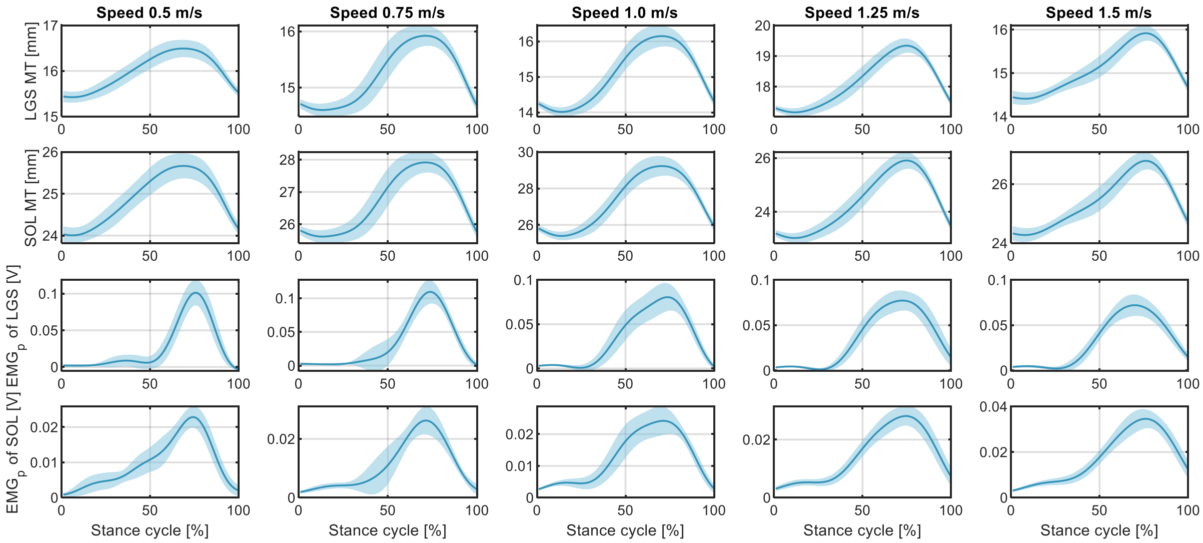

Figure S13 US imaging-derived MT and processed sEMG signals during the walking stance phase from Sub03

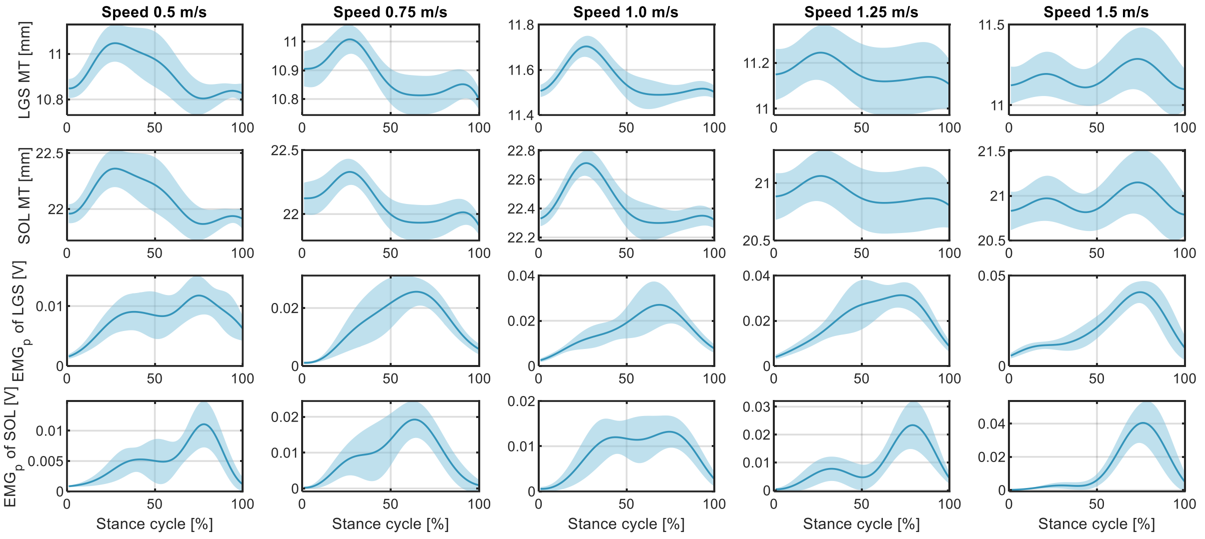

Figure S14 US imaging-derived MT and processed sEMG signals during the walking stance phase from Sub04

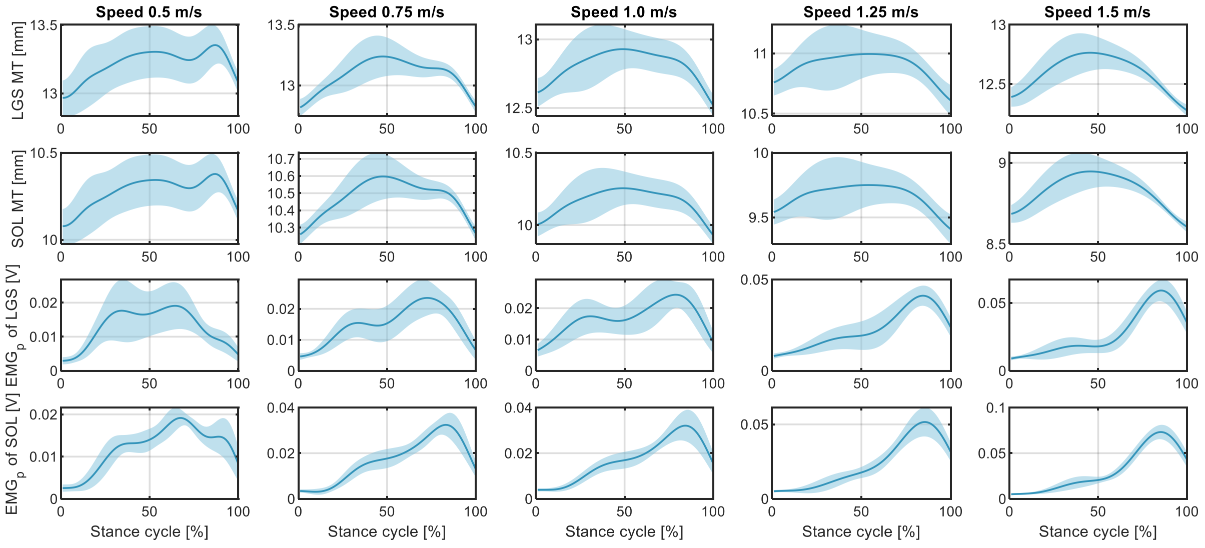

Figure S15 US imaging-derived MT and processed sEMG signals during the walking stance phase from Sub05

B. Muscle thickness tracking from US imaging by using UltraTrack

Take one representative participant walking at 1.00 m/s as an example, the UltraTrack toolbox interface, shown in Fig. S16 and Fig. S17, presents LGS and SOL muscle thickness tracking procedures and results without and with key-frame correction, respectively.

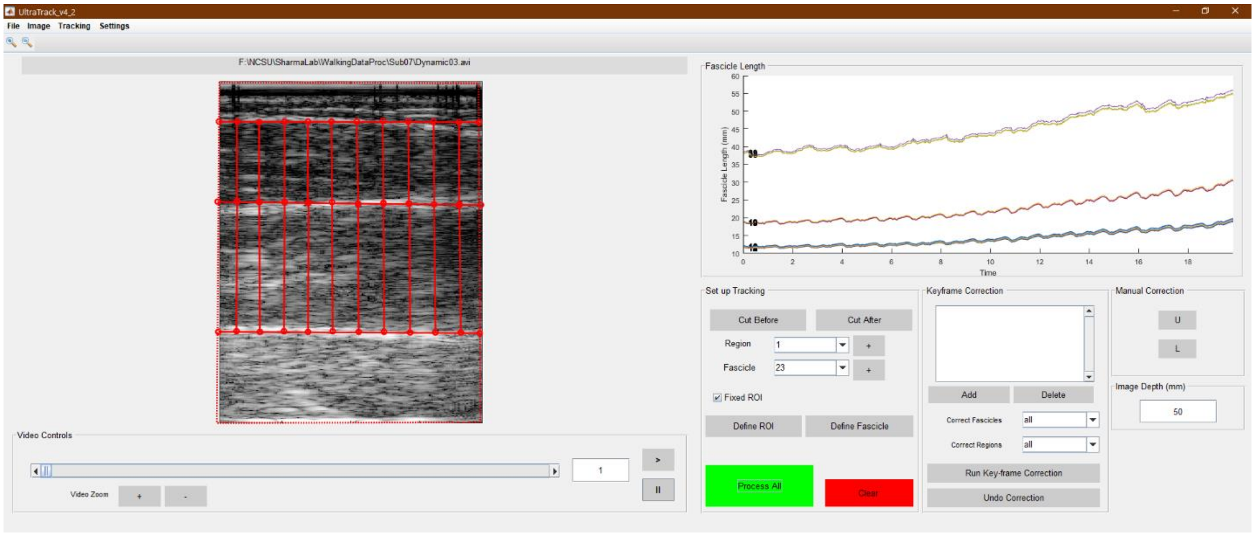

Figure S16 The Ultratrack toolbox interface with US imaging-derived MT tracking procedures and results without key-frame correction on Participant Sub07 walking at 1.00 m/s

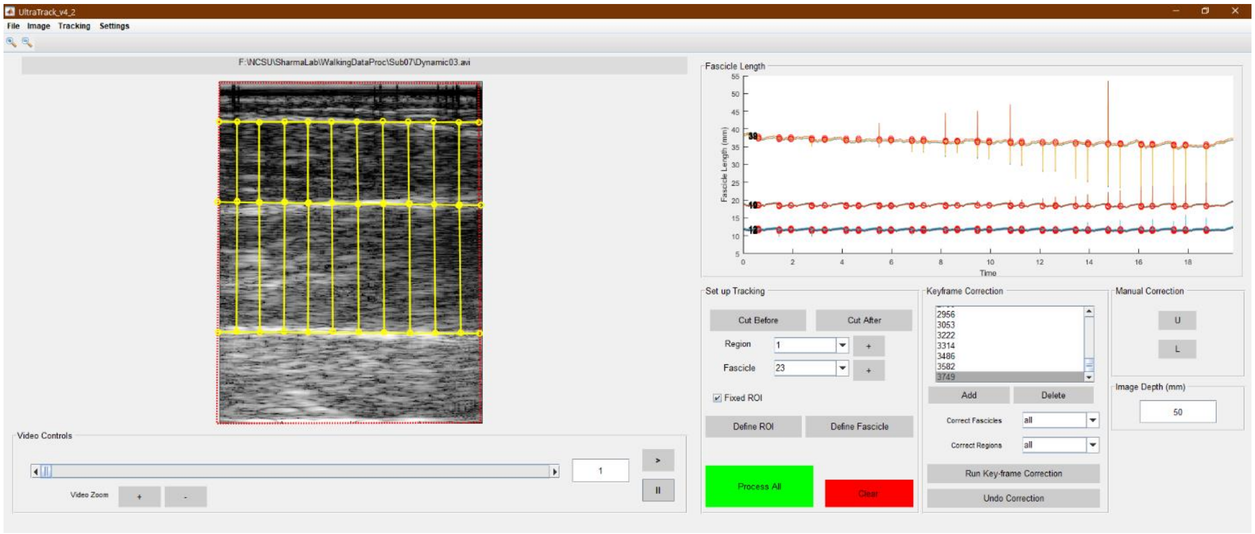

Figure S17 The Ultratrack toolbox interface with US imaging-derived MT tracking procedures and results with key-frame correction on Participant Sub07 walking at 1.00 m/s

C. Evaluation of HNMs prediction performance under single-speed modes and inter-speed mode

The HNMs prediction performance was evaluated through computing the RMSE and R^2 values between the predicted net PF moment and the measured net PF moment from inverse dynamics. Results of the RMSE and R^2 values in prediction from representative participants Sub01 to Sub05 are summarized in Table S-II to Table S-VI.

Table S-II HNMs prediction performance under single-speed modes and inter-speed mode on Sub01. (RMSE unit: Nm)

		Prediction scenarios									
Single-speed mode (0.50 m/s)		0.50 m/s		0.75 m/s		1.00 m/s		1.25 m/s		1.50 m/s	
		RMSE	R^2	RMSE	R^2	RMSE	R^2	RMSE	R^2	RMSE	R^2
	sEMG	12.15	0.954	25.60	0.808	39.97	0.684	54.23	0.585	55.38	0.569
	US	14.48	0.921	17.97	0.911	29.21	0.841	39.10	0.833	40.45	0.845
	sEMG+US	11.79	0.944	17.67	0.915	27.18	0.858	31.05	0.864	33.40	0.852
		Prediction scenarios									
Single-speed mode (0.75 m/s)		0.50 m/s		0.75 m/s		1.00 m/s		1.25 m/s		1.50 m/s	
		RMSE	R^2	RMSE	R^2	RMSE	R^2	RMSE	R^2	RMSE	R^2
	sEMG	17.49	0.917	14.33	0.955	20.16	0.955	28.27	0.939	44.42	0.938
	US	26.33	0.850	16.08	0.928	18.46	0.955	26.12	0.955	23.09	0.929
	sEMG+US	13.09	0.916	12.10	0.959	13.40	0.949	15.26	0.951	20.06	0.916
		Prediction scenarios									
Single-speed mode (1.00 m/s)		0.50 m/s		0.75 m/s		1.00 m/s		1.25 m/s		1.50 m/s	
		RMSE	R^2	RMSE	R^2	RMSE	R^2	RMSE	R^2	RMSE	R^2
	sEMG	25.24	0.875	30.27	0.922	12.08	0.962	22.83	0.947	17.53	0.937
	US	29.01	0.833	26.63	0.957	11.80	0.964	16.56	0.960	16.20	0.953
	sEMG+US	23.07	0.887	15.95	0.950	10.87	0.968	12.93	0.960	16.11	0.951
		Prediction scenarios									
Single-speed mode (1.25 m/s)		0.50 m/s		0.75 m/s		1.00 m/s		1.25 m/s		1.50 m/s	
		RMSE	R^2	RMSE	R^2	RMSE	R^2	RMSE	R^2	RMSE	R^2
	sEMG	34.37	0.816	28.86	0.882	16.90	0.932	12.24	0.962	15.68	0.95
	US	38.99	0.742	26.28	0.925	14.91	0.949	12.76	0.960	16.09	0.947
	sEMG+US	31.77	0.805	19.53	0.920	13.80	0.947	12.08	0.964	15.02	0.954
		Prediction scenarios									
Single-speed mode (1.50 m/s)		0.50 m/s		0.75 m/s		1.00 m/s		1.25 m/s		1.50 m/s	
		RMSE	R^2	RMSE	R^2	RMSE	R^2	RMSE	R^2	RMSE	R^2
	sEMG	43.31	0.772	32.71	0.892	20.46	0.936	13.81	0.958	16.18	0.940
	US	40.98	0.845	31.64	0.936	20.61	0.955	14.36	0.951	14.23	0.961
	sEMG+US	35.76	0.794	23.87	0.914	17.34	0.938	13.00	0.959	13.50	0.961
		Prediction scenarios									
Inter-speed mode (All speeds)		0.50 m/s		0.75 m/s		1.00 m/s		1.25 m/s		1.50 m/s	
		RMSE	R^2	RMSE	R^2	RMSE	R^2	RMSE	R^2	RMSE	R^2
	sEMG	16.84	0.917	11.79	0.959	14.83	0.941	16.40	0.956	20.17	0.930
	US	25.25	0.784	17.06	0.909	15.28	0.948	19.76	0.960	18.71	0.937
	sEMG+US	15.75	0.913	11.36	0.961	14.49	0.945	16.08	0.952	18.51	0.938

Table S-III HNMs prediction performance under single-speed modes and inter-speed mode on Sub02. (RMSE unit: Nm)

		Prediction scenarios									
Single-speed mode (0.50 m/s)		0.50 m/s		0.75 m/s		1.00 m/s		1.25 m/s		1.50 m/s	
		RMSE	R^2	RMSE	R^2	RMSE	R^2	RMSE	R^2	RMSE	R^2
	sEMG	6.83	0.907	9.30	0.891	18.50	0.628	32.23	0.276	51.74	0.106
	US	6.52	0.916	9.33	0.882	15.10	0.748	26.75	0.420	46.73	0.129
	sEMG+US	5.67	0.936	7.94	0.915	12.20	0.871	16.62	0.733	24.99	0.511
		Prediction scenarios									
Single-speed mode (0.75 m/s)		0.50 m/s		0.75 m/s		1.00 m/s		1.25 m/s		1.50 m/s	
		RMSE	R^2	RMSE	R^2	RMSE	R^2	RMSE	R^2	RMSE	R^2
	sEMG	8.61	0.880	8.02	0.915	16.95	0.699	32.70	0.337	56.05	0.160
	US	8.42	0.863	7.55	0.919	14.18	0.783	28.56	0.437	53.81	0.154
	sEMG+US	7.33	0.913	6.10	0.947	9.97	0.934	16.24	0.751	23.12	0.613
		Prediction scenarios									
Single-speed mode (1.00 m/s)		0.50 m/s		0.75 m/s		1.00 m/s		1.25 m/s		1.50 m/s	
		RMSE	R^2	RMSE	R^2	RMSE	R^2	RMSE	R^2	RMSE	R^2
	sEMG	15.46	0.799	10.80	0.898	7.43	0.937	15.34	0.774	25.57	0.683
	US	13.20	0.848	10.47	0.927	6.72	0.949	14.65	0.797	24.82	0.724
	sEMG+US	12.32	0.858	9.85	0.930	6.47	0.952	13.22	0.828	21.65	0.776
		Prediction scenarios									
Single-speed mode (1.25 m/s)		0.50 m/s		0.75 m/s		1.00 m/s		1.25 m/s		1.50 m/s	
		RMSE	R^2	RMSE	R^2	RMSE	R^2	RMSE	R^2	RMSE	R^2
	sEMG	17.64	0.796	10.40	0.897	9.60	0.934	12.22	0.858	13.28	0.871
	US	13.20	0.816	9.91	0.922	8.54	0.937	14.28	0.802	20.93	0.696
	sEMG+US	11.51	0.826	8.74	0.910	8.06	0.933	12.06	0.860	11.40	0.880
		Prediction scenarios									
Single-speed mode (1.50 m/s)		0.50 m/s		0.75 m/s		1.00 m/s		1.25 m/s		1.50 m/s	
		RMSE	R^2	RMSE	R^2	RMSE	R^2	RMSE	R^2	RMSE	R^2
	sEMG	25.93	0.311	23.53	0.742	27.40	0.726	28.65	0.651	11.23	0.881
	US	21.11	0.559	22.31	0.740	27.80	0.718	30.94	0.596	13.50	0.826
	sEMG+US	20.92	0.653	17.72	0.764	22.82	0.747	22.93	0.663	9.62	0.921
		Prediction scenarios									
Inter-speed mode (All speeds)		0.50 m/s		0.75 m/s		1.00 m/s		1.25 m/s		1.50 m/s	
		RMSE	R^2	RMSE	R^2	RMSE	R^2	RMSE	R^2	RMSE	R^2
	sEMG	10.02	0.830	8.99	0.890	9.74	0.922	16.19	0.754	21.90	0.652
	US	9.75	0.855	7.07	0.934	9.24	0.936	13.65	0.829	15.41	0.785
	sEMG+US	8.52	0.882	7.09	0.932	8.86	0.938	12.11	0.862	12.42	0.858

Table S-IV HNMs prediction performance under single-speed modes and inter-speed mode on Sub03. (RMSE unit: Nm)

		Prediction scenarios									
Single-speed mode (0.50 m/s)		0.50 m/s		0.75 m/s		1.00 m/s		1.25 m/s		1.50 m/s	
		RMSE	R^2	RMSE	R^2	RMSE	R^2	RMSE	R^2	RMSE	R^2
	sEMG	14.72	0.903	12.70	0.924	21.79	0.806	27.51	0.947	23.08	0.882
	US	15.33	0.881	14.10	0.944	27.38	0.929	49.98	0.874	30.06	0.854
	sEMG+US	13.31	0.935	11.89	0.957	17.91	0.900	19.91	0.949	17.42	0.909
		Prediction scenarios									
Single-speed mode (0.75 m/s)		0.50 m/s		0.75 m/s		1.00 m/s		1.25 m/s		1.50 m/s	
		RMSE	R^2	RMSE	R^2	RMSE	R^2	RMSE	R^2	RMSE	R^2
	sEMG	13.93	0.857	9.30	0.957	19.67	0.856	26.27	0.932	29.63	0.840
	US	15.37	0.844	11.50	0.931	15.52	0.925	36.51	0.920	35.31	0.720
	sEMG+US	11.86	0.893	6.99	0.977	14.87	0.918	21.27	0.967	25.88	0.790
		Prediction scenarios									
Single-speed mode (1.00 m/s)		0.50 m/s		0.75 m/s		1.00 m/s		1.25 m/s		1.50 m/s	
		RMSE	R^2	RMSE	R^2	RMSE	R^2	RMSE	R^2	RMSE	R^2
	sEMG	17.74	0.820	13.88	0.939	13.72	0.921	17.31	0.960	16.75	0.895
	US	17.51	0.831	13.16	0.932	12.37	0.935	31.44	0.915	31.96	0.723
	sEMG+US	12.97	0.883	8.51	0.963	9.47	0.961	13.63	0.932	17.56	0.924
		Prediction scenarios									
Single-speed mode (1.25 m/s)		0.50 m/s		0.75 m/s		1.00 m/s		1.25 m/s		1.50 m/s	
		RMSE	R^2	RMSE	R^2	RMSE	R^2	RMSE	R^2	RMSE	R^2
	sEMG	22.86	0.897	17.97	0.968	18.15	0.953	11.67	0.952	22.81	0.937
	US	50.90	0.836	62.81	0.934	64.91	0.929	15.28	0.921	78.57	0.792
	sEMG+US	18.74	0.946	14.39	0.947	13.67	0.927	11.47	0.955	13.91	0.928
		Prediction scenarios									
Single-speed mode (1.50 m/s)		0.50 m/s		0.75 m/s		1.00 m/s		1.25 m/s		1.50 m/s	
		RMSE	R^2	RMSE	R^2	RMSE	R^2	RMSE	R^2	RMSE	R^2
	sEMG	22.41	0.807	20.52	0.895	20.07	0.883	29.13	0.933	11.70	0.952
	US	20.57	0.692	20.65	0.791	28.64	0.799	38.91	0.825	12.04	0.945
	sEMG+US	13.05	0.870	11.87	0.935	13.49	0.937	19.39	0.891	11.01	0.953
		Prediction scenarios									
Inter-speed mode (All speeds)		0.50 m/s		0.75 m/s		1.00 m/s		1.25 m/s		1.50 m/s	
		RMSE	R^2	RMSE	R^2	RMSE	R^2	RMSE	R^2	RMSE	R^2
	sEMG	13.73	0.874	10.36	0.952	13.52	0.924	13.13	0.939	16.47	0.919
	US	18.18	0.808	13.10	0.918	16.19	0.915	17.25	0.906	30.34	0.741
	sEMG+US	12.07	0.914	8.15	0.967	11.37	0.950	12.36	0.959	14.11	0.930

Table S-V HNMs prediction performance under single-speed modes and inter-speed mode on Sub04. (RMSE unit: Nm)

		Prediction scenarios									
Single-speed mode (0.50 m/s)		0.50 m/s		0.75 m/s		1.00 m/s		1.25 m/s		1.50 m/s	
		RMSE	R^2	RMSE	R^2	RMSE	R^2	RMSE	R^2	RMSE	R^2
	sEMG	12.48	0.814	21.32	0.778	27.40	0.712	30.70	0.568	34.27	0.793
	US	14.73	0.735	34.58	0.664	49.58	0.600	40.25	0.375	55.70	0.139
	sEMG+US	11.72	0.834	19.46	0.796	23.63	0.758	29.66	0.639	33.09	0.815
		Prediction scenarios									
Single-speed mode (0.75 m/s)		0.50 m/s		0.75 m/s		1.00 m/s		1.25 m/s		1.50 m/s	
		RMSE	R^2	RMSE	R^2	RMSE	R^2	RMSE	R^2	RMSE	R^2
	sEMG	23.01	0.722	16.05	0.862	22.07	0.883	46.61	0.832	51.78	0.873
	US	36.07	0.666	20.20	0.780	24.98	0.849	65.18	0.770	61.13	0.544
	sEMG+US	21.14	0.713	15.52	0.870	15.45	0.883	18.92	0.829	26.64	0.876
		Prediction scenarios									
Single-speed mode (1.00 m/s)		0.50 m/s		0.75 m/s		1.00 m/s		1.25 m/s		1.50 m/s	
		RMSE	R^2	RMSE	R^2	RMSE	R^2	RMSE	R^2	RMSE	R^2
	sEMG	85.31	0.668	82.12	0.880	15.08	0.895	61.36	0.892	63.21	0.868
	US	66.96	0.639	52.80	0.768	17.15	0.853	24.66	0.823	43.97	0.641
	sEMG+US	22.22	0.671	14.75	0.879	13.62	0.905	15.38	0.891	26.76	0.872
		Prediction scenarios									
Single-speed mode (1.25 m/s)		0.50 m/s		0.75 m/s		1.00 m/s		1.25 m/s		1.50 m/s	
		RMSE	R^2	RMSE	R^2	RMSE	R^2	RMSE	R^2	RMSE	R^2
	sEMG	34.66	0.649	39.00	0.839	22.50	0.883	13.95	0.912	27.27	0.831
	US	34.99	0.598	24.87	0.693	20.06	0.813	17.14	0.874	32.57	0.766
	sEMG+US	25.42	0.645	17.18	0.838	15.16	0.883	13.94	0.912	27.04	0.832
		Prediction scenarios									
Single-speed mode (1.50 m/s)		0.50 m/s		0.75 m/s		1.00 m/s		1.25 m/s		1.50 m/s	
		RMSE	R^2	RMSE	R^2	RMSE	R^2	RMSE	R^2	RMSE	R^2
	sEMG	39.47	0.531	45.14	0.652	28.21	0.755	20.36	0.814	13.95	0.925
	US	34.71	0.590	53.10	0.487	31.48	0.653	22.43	0.760	14.26	0.921
	sEMG+US	22.12	0.595	24.83	0.663	22.27	0.761	20.27	0.813	13.40	0.929
		Prediction scenarios									
Inter-speed mode (All speeds)		0.50 m/s		0.75 m/s		1.00 m/s		1.25 m/s		1.50 m/s	
		RMSE	R^2	RMSE	R^2	RMSE	R^2	RMSE	R^2	RMSE	R^2
	sEMG	18.40	0.688	14.74	0.903	16.72	0.889	14.43	0.907	20.16	0.875
	US	26.55	0.562	25.76	0.624	22.03	0.745	16.96	0.868	21.31	0.839
	sEMG+US	18.44	0.691	14.46	0.904	16.41	0.891	14.46	0.908	20.02	0.876

Table S-VI HNMs prediction performance under single-speed modes and inter-speed mode on Sub05. (RMSE unit: Nm)

		Prediction scenarios									
Single-speed mode (0.50 m/s)		0.50 m/s		0.75 m/s		1.00 m/s		1.25 m/s		1.50 m/s	
		RMSE	R^2	RMSE	R^2	RMSE	R^2	RMSE	R^2	RMSE	R^2
	sEMG	10.12	0.879	14.22	0.841	14.65	0.803	20.37	0.823	21.56	0.812
	US	12.98	0.819	17.74	0.701	22.44	0.652	33.07	0.398	61.75	0.293
	sEMG+US	7.76	0.925	12.20	0.858	13.72	0.825	17.06	0.853	20.34	0.803
		Prediction scenarios									
Single-speed mode (0.75 m/s)		0.50 m/s		0.75 m/s		1.00 m/s		1.25 m/s		1.50 m/s	
		RMSE	R^2	RMSE	R^2	RMSE	R^2	RMSE	R^2	RMSE	R^2
	sEMG	13.88	0.780	10.05	0.898	21.65	0.913	23.98	0.801	22.95	0.895
	US	12.65	0.765	9.55	0.901	24.38	0.915	23.16	0.829	36.38	0.831
	sEMG+US	11.28	0.797	9.13	0.911	9.40	0.918	10.10	0.927	12.25	0.915
		Prediction scenarios									
Single-speed mode (1.00 m/s)		0.50 m/s		0.75 m/s		1.00 m/s		1.25 m/s		1.50 m/s	
		RMSE	R^2	RMSE	R^2	RMSE	R^2	RMSE	R^2	RMSE	R^2
	sEMG	16.10	0.743	10.59	0.877	9.99	0.909	18.47	0.872	37.44	0.583
	US	15.90	0.713	10.07	0.888	10.07	0.909	15.92	0.782	26.16	0.783
	sEMG+US	15.14	0.757	10.01	0.898	9.21	0.918	13.27	0.902	20.47	0.809
		Prediction scenarios									
Single-speed mode (1.25 m/s)		0.50 m/s		0.75 m/s		1.00 m/s		1.25 m/s		1.50 m/s	
		RMSE	R^2	RMSE	R^2	RMSE	R^2	RMSE	R^2	RMSE	R^2
	sEMG	25.10	0.637	17.75	0.863	18.20	0.866	9.07	0.932	12.40	0.893
	US	30.85	0.634	24.92	0.871	24.53	0.864	8.87	0.942	12.30	0.890
	sEMG+US	18.96	0.634	12.23	0.861	13.10	0.863	7.91	0.950	10.58	0.904
		Prediction scenarios									
Single-speed mode (1.50 m/s)		0.50 m/s		0.75 m/s		1.00 m/s		1.25 m/s		1.50 m/s	
		RMSE	R^2	RMSE	R^2	RMSE	R^2	RMSE	R^2	RMSE	R^2
	sEMG	19.38	0.596	14.51	0.819	16.43	0.805	10.71	0.929	7.67	0.957
	US	27.54	0.501	16.70	0.756	22.36	0.738	12.73	0.899	7.80	0.956
	sEMG+US	17.75	0.689	10.60	0.830	12.86	0.826	8.99	0.939	7.49	0.959
		Prediction scenarios									
Inter-speed mode (All speeds)		0.50 m/s		0.75 m/s		1.00 m/s		1.25 m/s		1.50 m/s	
		RMSE	R^2	RMSE	R^2	RMSE	R^2	RMSE	R^2	RMSE	R^2
	sEMG	13.02	0.796	9.98	0.904	9.90	0.913	9.61	0.936	10.57	0.929
	US	17.42	0.675	11.15	0.879	10.98	0.887	8.78	0.939	14.37	0.869
	sEMG+US	12.60	0.800	9.44	0.904	9.82	0.909	8.11	0.951	10.08	0.932

D. Additional results for supporting the discussion section

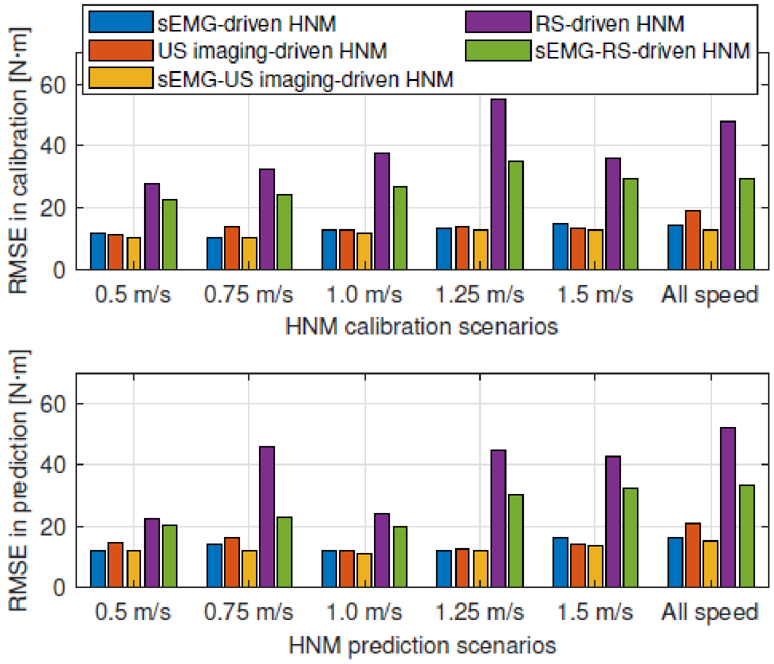

(a)

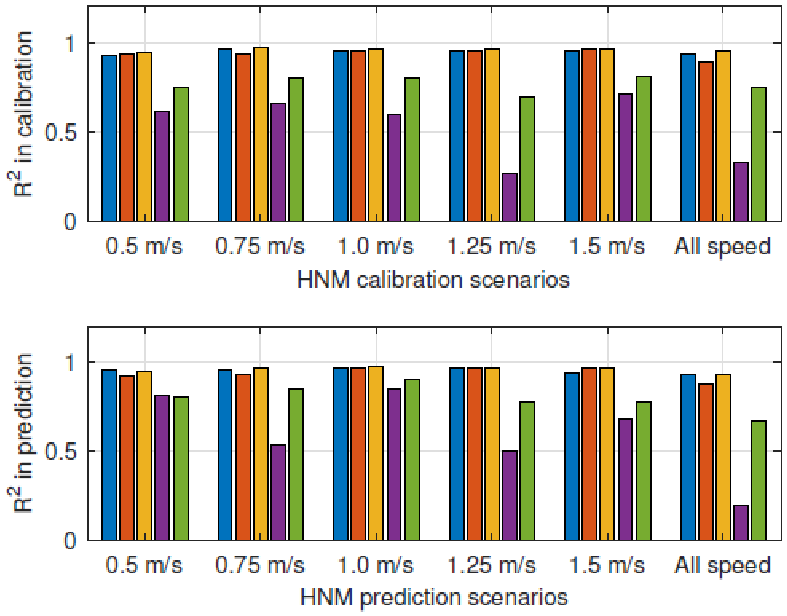

(b)

Figure S18: Exampled calibration and prediction performance on Participant Sub01 by using five HNM categories. The blue, red, orange, purple, and green bars represent the results by using the sEMG-, US imaging-, sEMG-US imaging-, random signal (RS)-, and sEMG-RS-driven HNMs under both single-speed modes and inter-speed mode. (a) Calibration *RMSE* and prediction *RMSE* values under different speed modes. (b) Calibration R-square and prediction R-square values under different speed modes.

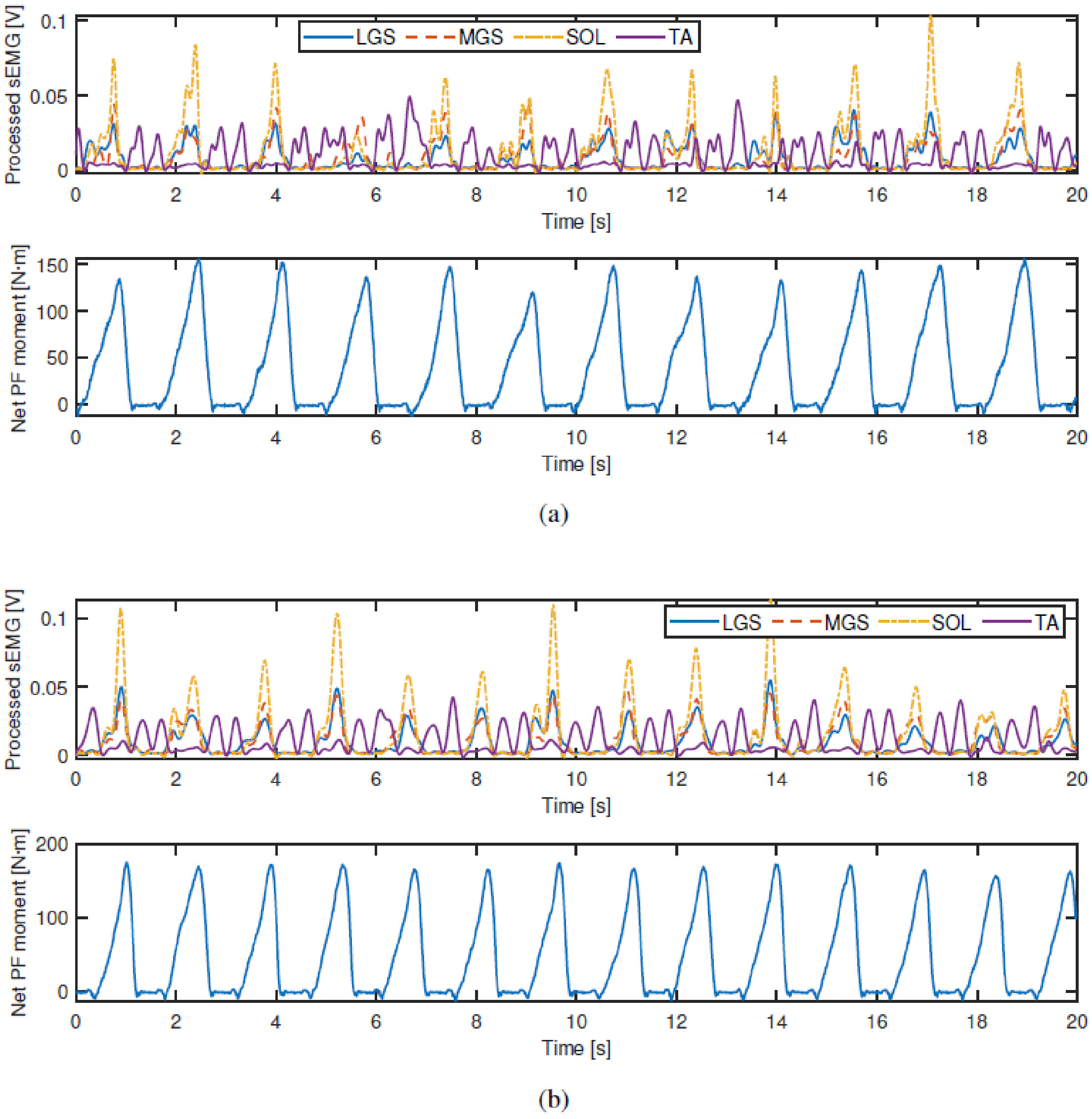

Figure S19: Exampled results of the ankle joint net moment and processed sEMG signals from LGS, MGS, SOL, and TA muscles throughout the recorded walking duration. Data are collected during the treadmill walking experiments on Sub01, where muscle activation levels from LGS and MGS muscles are comparable, and the activation level of the TA muscle is relatively small during the walking stance phase. (a) Walking speed at 0.5 m/s. (b) Walking speed at 0.75 m/s.

REFERENCES

[1] I. W. Charlton, P. Tate, P. Smyth, and L. Roren, “Repeatability of an optimised lower body model,” Gait & posture, vol. 20, no. 2, pp. 213–221, 2004.

[2] I. Campanini, A. Merlo, P. Degola, R. Merletti, G. Vezzosi, and D. Farina, “Effect of electrode location on emg signal envelope in leg muscles during gait,” J. Electromyogr. Kinesiol., vol. 17, no. 4, pp. 515–526, 2007.

[3] Q. Zhang, A. Iyer, K. Kim, and N. Sharma, “Volitional contractility assessment of plantar flexors by using non-invasive neuromuscular measurements,” in 2020 8th IEEE RAS/EMBS International Conference for Biomedical Robotics and Biomechatronics (BioRob). IEEE, pp. 515–520.
